# Rhamnogalacturonan‐II Dimerisation Reinforces Salt Resistance in Sugar Beet

**DOI:** 10.1111/pce.70457

**Published:** 2026-02-19

**Authors:** Shah Newaz Chowdhury, Lukas Pfeifer, Kim‐Kristine Mueller, Sazzad Hossain, Birgit Classen, Karl Hermann Mühling

**Affiliations:** ^1^ Institute of Plant Nutrition and Soil Science Kiel University Kiel Germany; ^2^ Department of Pharmaceutical Biology, Pharmaceutical Institute Kiel University Kiel Germany

**Keywords:** apoplastic fluid, arabinogalactan‐protein, *Beta vulgaris* L, boron, cell wall polysaccharides, cross‐linking, ion profile, pectic polysaccharides, rhamnogalacturonan‐II, salt stress

## Abstract

Salinity stress predominantly affects negatively charged cell wall polymers, for example, pectin. Excess Na^+^ ions interact physically and affect growth in stress‐sensitive plants. However, the salinity resistance of sugar beet cell walls remains unclear. To get a better understanding of cell wall assembly, we investigated arabinogalactan‐proteins (AGPs), extensins and pectic polysaccharides (homogalacturonan, rhamnogalacturonan‐I and rhamnogalacturonan‐II), in relation to underlying physiological mechanisms and growth expansion with low and adequate boron (B) under salinity. Findings revealed that salt stress affects AGPs and reduces cross‐linking of RG‐II, resulting in the softening of the sugar beet plant's cell wall. Adequate B compensates for plant growth by improving water flow into the cell, as indicated by the transpiration rate and stomatal conductance. In particular, the higher reduction of the Na^+^/Ca^2+^ ratio in the young leaves and apoplastic fluids and higher RG‐I content and dimeric RG‐II pectin (a key component of cell wall integrity) offered by adequate B, hint at protection against cell wall defects. However, no influence of B was detected for AGPs and extensins. This suggests that adequate B rescues cell wall integrity, thereby conferring strengthening and acid growth.

## Summary statement

Higher salinity disrupts plants' cell wall elements, for example, boron. Sugar beet with adequate boron shows salt resistance. As cell walls adapt to salinity, sugar beets with adequate boron offer sink leaves strength by increasing dimeric rhamnogalacturonan‐II and reducing Na^+^/Ca^2+^ ratio. The physiological response in relation to growth and ion profile strengthens our understanding of the physical interference of Na^+^ and plant resistance to salt stress.

## 1 Introduction

In the last few decades, limiting freshwater resources across most continents of the world have intensified environmental constraints, for example, soil salinity (Pitann et al. 2011; Musie and Gonfa 2023). At present, worldwide salt‐affected soil (EC > 4 dS/m) areas exceed 1.257 billion hectares, representing 9% of the total land area (FAO 2021), impacting various crop yield. Sodium chloride (NaCl) is a predominant soluble salt stressor for most salt‐sensitive species, and excessive intrusion at the threshold point impairs plants' ability to achieve tolerance levels. The decline in shoot and root growth in salt‐affected soils is primarily due to reduced water availability from osmotic effects, followed by ion toxicity within days to weeks later, resulting in mineral disturbances in plants (Tester 2003; Munns 2005; Munns and Tester 2008).

Glycophytes experience reduced growth and mortality at NaCl concentrations between 100 and 200 mM (Shabala 2013). In contrast, ‘salt‐loving’ halophytes such as *Festuca* sp., *Atriplex, Dichanthium, Salvodora* and *Tecticornia* thrive in saline conditions (Himabindu et al. 2016). Furthermore, among the cultivated crops, sugar beet (*Beta vulgaris* L.) shows significant growth in saline soil environments (Hossain et al. 2017), can tolerate salt concentrations from 100 to 300 mM of NaCl (Hossain et al. 2017; Pinheiro et al. 2018), and withstand exposure to 500 mM of NaCl for up to 7 days (Yang et al. 2012). The main reason has not yet been explored. However, leaf succulence, which enables sugar beet to store more water (Chiang et al. 2013) and salt, arises from the specialised parenchymatous tissue beneath the photosynthetically active chlorenchymatous tissue (Zeng et al. 2018) and is a key adaptation in the Amaranthaceous family. These surprising properties draw attention to studying specific cellular and molecular mechanisms involved in salt stress adaptation.

Extracellular space, that is, apoplast and cell wall, is crucial for plant leaf expansion and development through cellular ionic homoeostasis and regulating cell wall structure (Geilfus et al. 2015; Hocq et al. 2017). Plant cell walls are dynamic and complex structures, with many interrelated components determining their architecture and function. These components are constantly modified during development or in response to environmental stressors, acting as a first cell compartment of defence against stress (Silva et al. 2020; Pfeifer et al. 2022). The structural polysaccharides of the plant cell wall are a highly branched network of cellulose microfibrils, which are cross‐linked with hemicelluloses and embedded in a matrix of pectins (Doblin et al. 2010; Sørensen et al. 2011). However, this composition varies from monocots to dicots, between plant species, and among plant tissues where specific ions are involved in several steps for spatial orientation. In vascular plants, pectin fractions in cell walls contain a complex with the essential microelement boron (Hu and Brown 1994), which is vital for growth and development (Warington 1923; Wimmer et al. 2019). Boron has been found to either play a direct or an indirect role in maintaining the integrity of cell walls and membranes, synthesising carbohydrates, proteins and hormones, and is essential for cell elongation and growth (Brown et al. 2002; Wimmer et al. 2009; Voxeur and Fry 2014). The polysaccharides of the plant cell wall, particularly pectin rich in galacturonic acids (GalA), include homogalacturonans (HGs), substituted HGs like xylogalacturonans, rhamnogalacturonan‐I (RG‐I) and rhamnogalacturonan‐II (RG‐II). The primary pectic polymer, called HG, is initially highly methylesterified and undergoes selectively de‐methylesterification by pectin methylesterases (PME; Pelloux et al. 2007). Furthermore, pectate cross‐linking with Ca^2+^ forms ‘eggbox’ complexes (Grant et al. 1973), influencing wall porosity and the accessibility of the primary cell wall, which is crucial for plant cell function and interaction. Boron plays an important role in RG‐II, where two RG‐II monomers are covalently cross‐linked by boron. Boron participates in the formation of diester bonds with a specific apiose residue in each of two participating RG‐II domains in the primary cell wall. This reinforces divalent cations, for example, Ca^2+^ forming bridges through pectin crosslinking and stabilises the pectic network in the cell wall, which is crucial for the flexibility of the cell wall and structural integrity (Brown and Hu 1997; Ishii et al. 1999, 2002; O'Neill et al. 2004).

The hydroxyproline‐rich glycoproteins (HRGPs) comprise arabinogalactan‐proteins (AGPs, highly glycosylated), extensins (EXTs, moderately glycosylated) and proline‐rich proteins (PRPs, lightly glycosylated; Olmos et al. 2017; Johnson et al. 2018; Ma and Johnson 2023). In addition, a glycosylphosphatidylinositol (GPI) anchor at the AGP's C‐terminal side connects the protein to the plasma membrane in some cases (Silva et al. 2020). AGPs at the plasma membrane functionally act as a capacitor for extracellular Ca^2+^ or an essential partner in calcium signalling (Lamport and Várnai 2013; Lamport 2023) and are possibly also involved in the adaptation of seagrass cell walls to the marine environment (Pfeifer et al. 2020). NaCl stress resulted in the disruption of this homoeostasis. Initially, ionic stress has been shown to immediately arrest root growth (Shabala et al. 2016), but in the case of the shoot, this arrest occurs 10–14 days after the initiation of salt stress (Munns 2002). Moreover, Na^+^ exhibits increased toxicity in environments where numerous Na^+^‐permeable ion channels are responsive through passive uptake. The negatively charged polymers present in cell walls reversibly bind to cations such as Ca^2+^ and Na^+^. In the context of salinity stress, the accumulation of excess Na^+^ or an increase in the Na^+^/Ca^2+^ ratio, where Ca^2+^ is highly displaced by Na^+^ in the binding sites, results in the disruption of the structural matrix of the pectin ‘eggbox’ (O'Neill et al. 2004; Hocq et al. 2017; Zhao et al. 2020), thereby compromising cell wall integrity. Exogenous boron promotes cotton root cell wall cellulose, restoring ionic homoeostasis (Lu et al. 2023). It stimulates plasma membrane H^+^‐ATPase at the cellular level in the roots of *Hordeum vulgare*, thereby reducing K^+^ leakage (Qu, Havshøi, et al. 2024). It decreases cell membrane damage while increasing glycine betaine accumulation, thereby increasing water content (Karimi et al. 2018). The accumulation of soluble carbohydrates in leaves triggers osmoregulation by boron (Yousefi et al. 2020) and activates the phenylpropanoid biosynthesis pathway (Lu et al. 2025). At the gene expression level, a boron supply mitigates the negative effects of excessive salt by reducing Cl^‐^ transporters in sugar beet seedlings (Dong et al. 2021), while in rapeseed, Na^+^ retrieval via *BnaA2.HKT1* was facilitated (Hua et al. 2024). Recently, we found that sufficient boron supply enhances sucrose accumulation in the sink leaves, thereby facilitating mobility and augmenting the activities of the ASA‐GS cycle enzyme to protect against oxidative damage in sugar beet (Chowhury et al. 2025). In contrast, NaCl stress causes leaf apoplastic alkalinization and growth reduction (Geilfus 2017) by affecting cell wall polymers. Boron is instrumental for the cell walls, as a decrease in RG‐II pectin dimerisation causes growth inhibition (Baba et al. 2024; Jewaria et al. 2025). However, besides the wide range of ameliorating functions of boron, minimal research has addressed details regarding the physical interaction mechanisms and salt stress alleviation by boron.

Therefore, further investigations are necessary to ascertain the primary function of boron in pectin modification subsequent to alterations in the cell wall through physical interaction of Na⁺ in sugar beet plants. To resolve this question, we hypothesised that adequate boron in sugar beet plants enhances resilience against salinity stress by remodelling cell wall pectin and AGPs, thereby contributing to cell wall assembly, growth and development under salt stress.

## 2 Materials and Methods

### 2.1 Plant Materials, Growth Conditions and Treatments

The methodology described below is also included in the supplement as a schematic diagram (Supporting Information: Figure S1). Seeds of sugar beet (*Beta vulgaris* L. cv. Strube salz 3) were obtained from Strube Research GmbH & Co. KG, Söllingen, Germany and grown hydroponically under a controlled environment in a climatic chamber. Conditions, viz., photoperiod 14:10 h (light/dark cycle); relative humidity 60% and temperature 20°C/15°C (day/night); light intensity 350 μmol photon m^−2^ s^–1^ (recorded by a light metre, Li‐198, Lincoln, USA), for the whole study period as previously described by Chowdhury et al. (2024). Briefly, uniform‐sized seeds were selected and soaked in ultrapure water (18.25 MΩ, ddH_2_O) with aeration for 12 h. Then, germinated on a filter paper moistened with ultrapure water (18.25 MΩ, ddH_2_O) at 20°C for 4 days in darkness and finally exposed to light. Two uniform growth‐sized 12‐day‐old sugar beet seedlings were transferred to 2.5 L plastic pots containing a quarter‐strength of the nutrient solution (NS). Followed by Hoagland and Arnon (1950), hydroponics NS was used with minor modifications: 1.0 mM KNO_3_, 2.0 mM Ca(NO_3_)_2_, 0.5 mM MgSO_4_, 0.1 mM CaCl_2_⋅2H_2_O, 0.50 mM (NH_4_)H_2_PO_4_, 2 μM MnSO_4_, 0.5 μM ZnSO_4_, 0.3 μM CuSO_4_, 0.1 μM NH_4_Mo_2_O_24_ and 40 μM Fe‐EDTA. NS concentration was progressively increased to half strength on the third day and full strength beginning the second week after the transplanting to prevent an osmotic shock reaction. Pots with NS were continuously oxygenated to the roots and changed every 2 days to replenish depleted nutrients. Based on the pilot study, two conditions of boron, such as low boron (LB; 0.25 μM) and adequate boron (AB; 25 μM) as $H_{3}^{10}BO_{3}\;$(99% ^10^B atom abundance, Sigma‐Aldrich, USA), started after transplanting and continued until harvesting. Under control conditions, both boron‐treated plants grew for 28 days, and then salt (NaCl, 0 and 300 mM) was carried out for 14 days with LB and AB conditions (Supporting Information: Figures S2 and S3). Up‐salting was done daily with 50 mM NaCl to avoid acute osmotic shock and continued until the concentration reached 300 mM (Hossain et al. 2017). Beginning to the end of the study, to avoid boron contamination in every single step, from solution preparation to harvesting and analysis, double‐deionised water (18.2 MΩ, ddH_2_O) and plastic were used. There were four identical groups of plant cultivations with four replicates for each treatment (*n* = 4).

One set of plant cultivation was used for recording dry mass and nutrient status and the transpired volume of water at harvesting, followed by Dinh et al. (2021). Briefly, the daily water loss during this period was measured by weighing each pot before changing the NS. The mean evaporation rate was evaluated using pots containing nutritional solutions devoid of plants. The water transpiration from each pot was determined by subtracting the water loss from the pots without plants from those with plants every 48 h for a period of treatment application. Apoplastic and symplastic fluids were collected from young leaves of the second set of experiments, young leaf area and apoplastic pH from the third set. fouth set experiments to do biochemical studies, where samples were freshly separated from the base pairs: first, second and third pairs represented old leaves based on true leaves, while the fourth and fifth pairs represented young leaves (Chowdhury et al. 2025). After separating leaves and roots, samples were washed three times with ddH_2_O (18.2 MΩ), blotted with tissue paper, and immediately frozen in liquid nitrogen by wrapping them in aluminium foil, then stored at −80°C. The experiment was repeated three times, maintaining similar climatic conditions in the climatic chamber in winter, with constant results from 2022 to 2024.

### 2.2 Plant Growth and Apoplastic pH

The roots and leaves were harvested separately from the first set of experiments and then rinsed three times with ddH_2_O (18.2 MΩ) to eliminate any adhering nutrients. They were subsequently dried using tissue paper. Fresh weights (g) of the plant samples were measured. After being placed in an oven for 3 days (72 h) at 65°C until reaching a stable weight, the dry matter weight (g) of the shoots and roots was determined. The area of young leaves in each plant was measured by using a smartphone app (Petiole LTD 2022) after harvesting. Leaf apoplastic pH measurement in situ was done according to Geilfus and Mühling (2011) in the Mühling lab using young leaves. In situ leaf apoplastic pH was measured using the 488‐dextran conjugated ratio metric fluorescent pH indicator dye Oregon Green (OG) at a concentration of 30 μM (Invitrogen GmbH, Darmstadt, Germany; Geilfus and Mühling 2011). Fluorescence images were captured as a single point measurement with an iMIC inverted microscope (iMIC, FEI Munich GmbH, now Thermo Fisher) at wavelengths of 440 and 490 nm (pH‐insensitive and sensitive). The F490/F440 fluorescence ratio was calculated for each pixel to determine pH. The obtained ratio values were converted into data on leaf apoplastic pH using in vivo calibration. The calibration data were fitted with a sigmoidal curve using the Boltzmann fit. Four biological replicates were performed, with several technical replicates.

### 2.3 Water Relations and Plant Photosynthesis

A portable open‐flow gas exchange system (LI‐6400XT, Li‐COR Biosciences Inc.) was used to measure water‐related attributes, for example, transpiration rate (*Tr*) and photosynthetic attributes such as stomatal conductance (*g*_*s*_) and net photosynthetic rate (*A*_*n*_), in light‐adapted plants as described by Sagervanshi et al. (2022). For the measurement, excluding the midrib from the base, a third pair of fully expanded leaves was selected between 10:00 a.m. and 2:00 p.m. on the first day (1), in the middle (2), and on the final day at harvesting (3) after up‐salting (*n* = 4 per treatment). Measurements were carried out by placing leaves in a 2 × 3 cm leaf chamber at 500 μmol m^−2^ s^−1^ photosynthetic photon flux density (PPFD) using the LED light source (LI‐6400‐02, LI‐COR Biosciences); the flow rate was set to 500 μmol s^−1^ and the CO_2_ concentration of the incoming air was regulated to 405 μmol mol^−1^ by CO_2_ injection (LI‐6400‐01, LI‐COR Biosciences). The temperature in the measuring cuvette was set to 20^◦^C. Following this, a nondestructive method was employed to estimate chlorophyll content using a portable chlorophyll metre (SPAD‐502, Minolta, Marunouchi, Japan) on the first day (1), at the midpoint (2) and during harvesting (3) after up‐salting (*n* = 4 per treatment) on the third pair of fully expanded leaves. Values were collected from five to eight points on the selected leaf, and the mean value was used for each treatment.

### 2.4 Collection of Washing Fluids and Elemental Analysis

The second set of experiments was conducted to collect the apoplastic washing fluid and isolate the symplastic fluid from undamaged young leaves, in the Mühling lab, followed by the infiltration‐centrifugation method (Mühling and Sattelmacher 1995; Masood et al. 2012; Sagervanshi et al. 2022). Briefly, the young leaves from the fouth and fifth pairs were collected and rinsed with Milli‐Q water (18.2 MΩ). Then, the intact leaves were gently placed into a 100 mL plastic beaker filled with 70 mL of Milli‐Q water under vacuum for 5 min. Subsequently, tissue paper was used to blot dry the leaves, which were centrifuged at a force of 245 g for 15 min at a temperature of 6°C using swing‐out buckets to separate the washing fluid. The first fluid that came out was the apoplastic washing fluid. Afterwards, the remaining leaf tissue samples were rapidly frozen in liquid nitrogen (−80°C), subsequently thawed at ambient temperature, and again centrifuged at a force of 703 g for 15 min at a temperature of 4°C. Then, the fluid was collected as symplastic fluid. Both fluids were stored at −80°C until elemental analysis.

### 2.5 Extraction and Elemental Analysis in Dry Matter

Elemental analysis was performed using a grinder to fine‐powder ground tissue samples (Cyclotec 1093, Foss Tecator, Höganäs, Sweden). The dried plant materials were subjected to digestion using 10 mL of 69% HNO_3_ (ROTIPURAN Supra for ICP, 69%) in a closed‐vessel 1800‐watt microwave digestion system (MARS 6 Xpress, CEM Corporation, Matthews, NC, USA), with the specified parameters as detailed by Chowdhury et al. (2024). Subsequently, the processed samples were diluted with a 2% solution of nitric acid (HNO_3_) to a volume of 100 mL with conductivity Milli‐Q water (18.2 MΩ) and then kept at a temperature of 4°C until further investigation.

In both tissue and fluids, the concentration of boron (^10^B) was determined by an inductively coupled plasma mass spectrometer (ICP‐MS; Agilent 7700, Agilent Technologies Inc., Santa Clara, CA, USA), and Na^+^, K^+^ and Ca^2+^ were determined by inductively coupled plasma optical emission spectrometry (ICP‐OES; Agilent 5800, Agilent Technologies Inc., USA) in the Mühling lab.

### 2.6 Isolation of Cell Wall Fractions

The grounded and freeze‐dried leaf and root material was used to gain different cell wall fractions. The aqueous extract (AE) was extracted with doubled distilled water (ddH_2_0) after a two‐times pre‐extraction with 70% (V/V) acetone, both in a ratio 1:10 (w/V), according to Pfeifer et al. (2023). The resulting insoluble plant pellet was separated by a vacuum pump and used to obtain a pectic fraction (AmOx), extracted with 0.2 mol L^‐1^ ammonium oxalate in a 1:100 ratio (*w/V*), and a further pectic fraction (Na_2_CO_3_), via 100 mL 3% (w/V) sodium carbonate solution at 70°C [see modified method of O'Rourke et al. (2015), described in Pfeifer et al. (2023)].

### 2.7 Analysis of Monosaccharides and the Amino Acid Hydroxyproline (Hyp)

The monosaccharides of the cell wall fractions were determined following the modified method of Blakeney et al. (1983), described in Mueller et al. (2023). After acetylation of the neutral monosaccharides, these were identified and quantified by gas chromatography (GC) with flame ionisation detection (FID) and mass spectrometry detection (MSD): GC + FID: 7890B; Agilent Technologies, USA; MS: 5977B MSD; Agilent Technologies, USA; column: Optima‐225; Macherey‐Nagel, Germany; 25 m, 250 μm, 0.25 μm; helium flow rate: 1 mL min^−1^; split ratio 30:1. Peak separation was accomplished by a temperature gradient (initial temperature 200°C, subsequent holding time of 3 min; final temperature 243°C with a gradient of 2°C min^−1^).

Monosaccharide analysis of the AmOx fraction (including neutral and acidic monosaccharides) was determined additionally following the workflows of Bleton et al. (1996) and Doco et al. (2001) with modifications. In brief, 2 mg of the sample was methanolized (1 mL of methanolic‐HCl produced in situ by adding acetyl chloride to water‐free methanol, 1:6) at 80°C for 24 h. After removal of solvents under a stream of nitrogen, the silylating agent (*N*‐trimethylsilyl imidazole + anhydrous pyridine) was added and incubated under a nitrogen atmosphere for 2 h at 80°C. The sample was analysed by GC‐MS within 12 h after derivatisation on the above‐mentioned GC‐MS + FID system using a SBP‐1 column (Supelco®, Merck KGaA, Darmstadt, Germany, 30 m, 250 µm, 0.25 µm, helium flow rate: 1 mL min^−1^; split ratio 30:1; temperature gradient as described in Doco et al. 2001). Peak identification was performed using standard sugars and response factors relative to *myo*‐inositol. RG‐I contents were inferred absolute mass of detected Rha and equimolar GalA contents. HG contents are calculated from the remaining absolute GalA contents.

The Hyp content was determined photometrically at 558 nm (UV‐1280, Shimadzu, Kyōto, Japan) according to the method of Stegemann and Stalder (1967) with slightly Modifications (Mueller et al. 2023) by linear regression analysis (4‐hydroxy‐L‐proline standard).

### 2.8 Radial Gel Diffusion

The AGP contents in the AE were determined using the methodology described by Castilleux et al. (2020). In brief, standard solutions of Gum Arabic in ddH_2_0 (Sigma‐Aldrich Chemie GmbH, Taufkirchen, Germany; negative control, 0.0625, 0.125, 0.25, 0.5, 1.0 mg mL^−1^) as well as samples (concentration 10 mg mL^−1^ for old and young leaves samples, 3.3 mg mL^−1^ for root samples) were filled into cavities in an agarose gel containing β‐glucosyl Yariv reagent (βGlcY, synthesised in the Classen lab following Yariv et al. 1962). After 2 days of incubation, the diameters of the circular halos formed around the cavities were measured using ImageJ (version 2.1.0/1.53c). Concentration of AGPs in the samples were determined by linear regression of the Gum Arabic standards.

### 2.9 Polyacrylamide Gel Electrophoresis (PAGE)

Characteristics in RG‐II content, as well as monomer and dimer ratios were analysed using the PAGE workflow described by Chormova et al. (2014). Approximately 50 mg of the freeze‐dried and pulverised plant samples were extracted with 1.5 mL ethanol 80% (V/V) in a VWR Star‐Beater (25 Hz, 2 min, stainless steel beads) with subsequent centrifugation (10.000 *g*, 10 min, 4°C, Eppendorf centrifuge 5417 R, Eppendorf SE, Hamburg, Germany). Following that, the samples were demethylated with 1 M Na_2_CO_3_ for 16 h at 4°C. The samples were washed with ddH_2_O until the washing solution had a neutral pH. After the last centrifugation step, residual water was removed by a final washing with aceton. The dried alcohol‐insoluble and demethylated samples (AIR) were treated with endopolygalacturonase (0.5 U/mg AIR; E‐PGALPC, Megazyme Ltd., Wicklow, Ireland) in 50 mM acetate buffer (pH 5.2) for 16 h at 40°C. 8 µL of solubilised pectins and 2 µL of sample buffer (0.63 M TRIS‐HCl, pH 8.8, containing 0.25% w/v bromophenol blue and 50% glycerol) were loaded into the cavities of a 23.3% polyacrylamide gel. The gels were silver‐stained as described by Chormova et al. (2014), and band intensities were quantified using ImageJ (version 2.1.0/1.53c). Three concentrations of wine RG‐II (RG‐II dimers, isolated in the Classen lab) as well as hydrolysed wine RG‐II (mainly RG‐II monomers) were used as calibration standards for regression analysis.

### 2.10 Statistical Analysis

The experimental data were statistically analysed using a two‐way analysis of variance (ANOVA). The analysis was performed using R Studio (version 4.2.2) R Core Team (2022). Means were then compared using Tukey's HSD test, facilitated by the Agricolae package, at *p* ≤ 0.05. Graphs and plots were generated using GraphPad Prism 9 (version 9.5.0, GraphPad Software, San Diego, California, USA).

The cell wall biochemistry data were tested for normal distribution with the Shapiro–Wilk and Kolmogorov–Smirnov normality tests included in R Studio (version 2024.12.1 + 563). As some data were not normally distributed (*p* ≤ 0.05), the built‐in nonparametric Kruskal–Wallis rank sum test was performed with subsequent Dunn‐Test (from the rstatix package) for multiple pairwise comparison.

Results from the acetylation analysis were compared using principal component analysis (PCA). The built‐in functions as well as the ggfortify package were used to generate scaled and centred biplots showing underlying influencing factors within the datasets.

## 3 Results

### 3.1 Influences of Salt and Boron on Growth

#### 3.1.1 Dry Matter Yield (Biomass)

**Figure 1:**
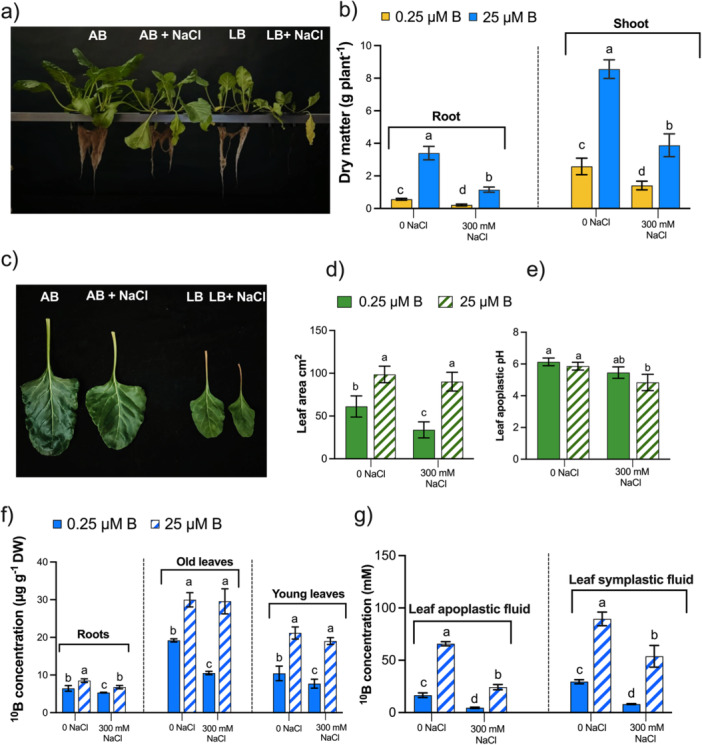
Boron (B) accelerates the growth of *Beta vulgaris* plants under high salt stress. (a) The growth phenotype, (b) root, and shoots dry matter, (c) young leaf growth differences, (d) leaf area of young leaves, (e) young leaf apoplastic pH, (f) boron (^10^B) concentrations in roots, old, young leaves, (g) young leaves apoplastic and symplastic fluids of sugar beet under low (0.25 μM, LB) and adequate (25 μM, AB) levels of boron after NaCl stress (0/300 mM NaCl). Sugar beet plants were cultivated in Hoagland's nutrient solution for 28 days with LB and AB conditions. Then, NaCl stress conditions (0/300 mM NaCl) were applied for another 14 days, containing LB and AB. Values are means ± SD with four biological replicates (*n* = 4) at *p* < 0.05 (Tukey's HSD test).

In order to explore the significance of boron in enhancing salt resistance in sugar beet in hydroponic growth systems, we determined the dry biomass of shoots and roots by supplying low (LB; 0.25 μM ^10^B) and adequate boron (AB; 25 μM ^10^B) after 14 days of high saline condition (300 mM NaCl; Figure 1a,b, Supporting Information: Figure S3a,b). The dry weight of shoots and roots was intensely inhabited by LB supply in both control and salt stress compared to the AB (Figure 1a,b). Both shoots and root's dry weight increased significantly in AB supply in the absence or after exposure to 300 mM salt compared to LB plants. Salt‐grown sugar beet plants with AB augmented shoot and root dry biomass by 2.7 and 5.4‐fold compared to LB, respectively (Figure 1b).

#### 3.1.2 Leaf Area

The leaf area of sugar beet plants was significantly influenced by boron supply in both high saline and nonsaline conditions (Figure 1c,d). Compared to LB, the leaf area of AB increases 2.7‐fold in young leaves that grow following salt initiation (Figure 1d). Plants treated with AB under salinity conditions similar to those in nonsaline conditions had an increased leaf area. By contrast, LB reduced the area of young leaves under saline conditions compared to nonsaline conditions.

#### 3.1.3 Leaf Apoplastic pH

The influences of salt and boron on apoplastic pH were assessed in young leaves grown under 0 and 300 mM NaCl stress conditions (Figure 1e). The mean fluorescent ratio was recorded on the young fouth leaves of each plant, with two points recorded on each leaf. This resulted in a total of eight points for both boron‐treated plants under nonsaline and saline conditions (*n* = 4). Slightly acidification of the leaf apoplast obtained by LB‐treated salt‐stressed sugar beet plants, however, was pronounced in AB‐treated plants. Furthermore, no significant influence of boron was demonstrated under nonsalt conditions.

### 3.2 Influences of Salt and Boron on Boron (^10^B) Concentration and Accumulation

Under the same treatment conditions, the concentration of boron (^10^B) in both leaves was significantly higher than in the roots, as depicted in Figure 1f,g. Adequate application of boron (25 μM ^10^B) under 0 mM NaCl and 300 mM NaCl stress increased boron (^10^B) concentrations in the roots, old and young leaves, leaf apoplastic and symplastic fluids (Figure 1f,g). However, under 14 days of 300 mM NaCl salt with LB, there was a reduction in boron (^10^B) concentration of 1.3‐, 2.8‐, 2.5‐, 5.4‐ and 6.6‐fold, respectively, compared to the AB‐supplied plants. Notably, the presence of AB in salt‐grown sugar beet plants resulted in an enhanced boron (^10^B) concentration compared to nonsaline‐grown LB plants in both leaves and fluids (apoplastic and symplastic). Conversely, root boron (^10^B) concentration remained unaltered between saline‐grown AB and nonsaline‐grown LB (Figure 1f). While there was a notable increase in boron (^10^B) accumulation in both leaves and roots recorded (Supporting Information: Figure S4c,d).

### 3.3 Influences of Salt and Boron on Water Relations and Photosynthesis

**Figure 2:**
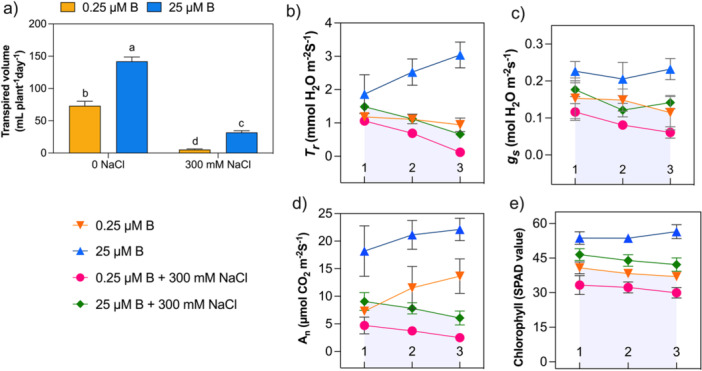
Influences of boron on water relations and photosynthetic attributes of high salt‐grown sugar beet plants. 1st day at 300 mM NaCl (1), at the middle (2) and harvesting (3). (a) Transpired volume of water at harvesting, (b) *T*_*r*_: Leaf transpiration rate, (c) *g*_*s*_, leaf stomata conductance, (d) *A*_*n*_, Net photosynthesis rate, (e) Chlorophyll (SPAD value) of sugar beet under low (0.25 μM, LB) and adequate (25 μM, AB) levels of ^10^B after NaCl stress (0/300 mM NaCl). Sugar beet plants were cultivated in Hoagland′s nutrient solution for 28 days with LB and AB conditions. Then, NaCl stress conditions (0/300 mM NaCl) for another 14 days, containing LB and AB. Values are means ± SD with four biological replicates (*n* = 4) at *p* < 0.05 (Tukey′s HSD test). [Color figure can be viewed at wileyonlinelibrary.com]

Our results depicted that the transpired volume of water was significantly influenced by the adequate presence of boron (AB; 25 μM B) in both control and high salt stress groups of sugar beet plants (Figure 2a). On average, AB‐treated plants without/exposure to NaCl stress transpired 2.0‐ and 6.1‐fold more water than long‐time LB‐supplied sugar beet plants. Under high salt stress (300 mM NaCl), transpiration and stomatal conductance rate were promoted with AB supplementation, in contrast to the results obtained with LB (Figure 2b,c). In detail, under 300 mM NaCl stress, the transpiration rate and stomatal conductance decreased with a delay of the mid‐points (2) after up‐salting in both LB and AB‐treated plants. Long‐time LB plants showed a gradual decline at harvesting of observation, which was interestingly uplifted in AB plants (Figure 2b). In the control group, at the end of the observation, transpiration rate and stomatal conductance declined in the LB plants compared to the AB plants.

The photosynthesis rate and chlorophyll (SPAD value) confirmed the ameliorative effects of salt stress by the presence of AB (Figure 2d,e). On the contrary, the photosynthetic activity and SPAD value detected declined in the LB with both 0 NaCl or exposure to 300 mM NaCl‐treated conditions. Our observation decapitated that under salt conditions from the first day to harvest, both the deprivation and adequate supply of boron dropped down both values. However, LB experienced a continuous and significant decline, whereas AB decreased slowly.

### 3.4 Influences of Salt and B on Na^+^, K^+^ and Ca^2+^ Concentration and Ratio

**Figure 3:**
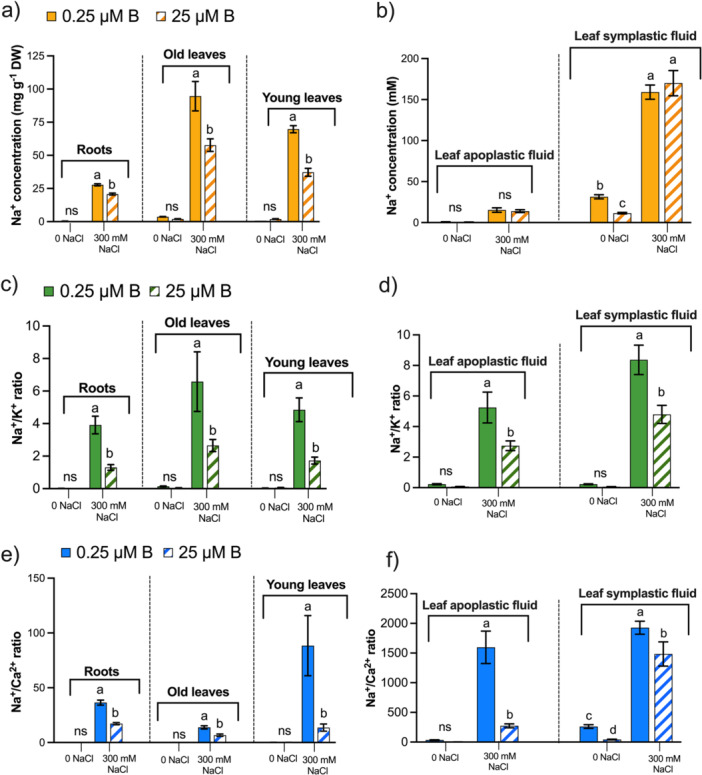
Boron creates differences in ion concentrations and ratios in high‐salt‐grown sugar beet plants. The concentration of Na^+^ (a–b), ratio of Na^+^/K^+^ (c–d) and Na^+^/Ca^2+^ (e–f) in roots, old, young leaves, young leaves apoplastic and symplastic fluids of sugar beet under low (0.25 μM ^10^B, LB) and adequate (25 μM ^10^B, AB) levels of B after NaCl stress (0/300 mM NaCl). Sugar beet plants were cultivated in Hoagland′s nutrient solution for 28 days with LB and AB conditions. Then, NaCl stress conditions (0/300 mM NaCl) were applied for another 14 days, containing LB and AB. Values are means ± SD with four biological replicates (*n* = 4) at *p* < 0.05 (Tukey′s HSD test). [Color figure can be viewed at wileyonlinelibrary.com]

The concentration of Na^+^ increased markedly in NaCl‐treated sugar beet plants at both boron conditions (LB/AB) compared to their respective controls, with significant increases subjected to LB with 300 mM NaCl stress (Figure 3a, Supporting Information: Figure S3c,d). In the roots, Na^+^ concentration was 1.3‐fold reduced in the presence of AB supply compared with LB‐treated plants under 300 mM NaCl (Figure 3a). A similar finding was also observed for both old and young leaves. Although no significant changes were reported in the leaf apoplastic and symplastic fluids (Figure 3b).

NaCl stressor reduced K^+^ and Ca^2+^ concentrations in the AB and LB‐treated group compared to the control group of sugar beet plants (Supporting Information: Figure S5a–d). However, K^+^ and Ca^2+^ concentrations were significantly higher in AB‐grown plants than in LB with high salt (300 mM NaCl) in the roots, both old and young leaves, apoplastic and symplastic fluids (Supporting Information: Figure S5a–d). Changes were 2.3‐, 1.5‐, 1.4‐, 1.7‐ and 1.9‐fold for K^+^ and 1.3‐, 3.4‐, 1.6‐, 5.3‐ and 1.4‐fold for Ca^2+^, respectively. Similar effects were reported for those without NaCl‐treated plants. An AB level of boron has been shown to lead to a reduction in Na^+^/K^+^ and Na^+^/Ca^2+^ ratios in both tissues (leaves and roots) and, in the fluids (apoplastic and symplastic; Figure 3c–f, Supporting Information: Figure S3e–h). The results reported that the replacement of Ca^2+^ by Na^+^ is much higher in young leaves than in the old leaves and roots under LB, while this displacement is lower in AB‐treated salt‐stressed sugar beet plants (Figure 3e). Similarly, higher Ca^2+^ by Na^+^ replacement was also observed in apoplastic fluids than in symplastic fluids of the young leaves in LB‐treated plants under higher salinity (Figure 3f).

### 3.5 Influence of Salt and Boron on AGPs

#### 3.5.1 Yield

The yield of water‐soluble polysaccharides including AGPs (AE) varied between 1.4% and 6.4% of the dry plant weight and was highest in old leaves (Supporting Information: Figure S6). In general, there was a trend to higher yields in salt‐grown plants, and all organs showed the highest amounts during cultivation with salt and adequate boron.

#### 3.5.2 Composition

The monosaccharide composition of all AE fractions is shown in Supporting Information: Figure S7 and Table S1a–c. Ara and Gal were the dominating neutral monosaccharides in AE of all organs under all conditions and comprised together between 61.1% and 84.2%, thus reflecting that mainly AGPs are present in AE fractions. Further monosaccharides present in minor amounts were Glc, Rha, Xyl and Man, sometimes accompanied by traces of Fuc. The content of Xyl was higher in young leaves (9.1%– 13.2%) compared to old leaves (3.3%–9.0%) and roots (2.2%– 5.1%).

**Figure 4:**
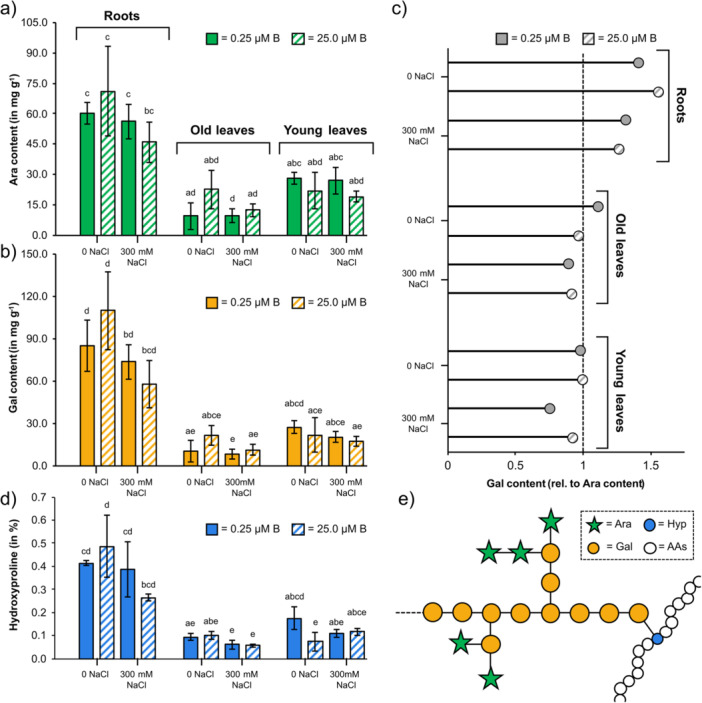
Amounts of arabinose (Ara), galactose (Gal) and hydroxyproline (Hyp) in water‐soluble fractions from roots and leaves of sugar beet under different salt and boron conditions. (a) Ara content as determined by *per*‐acetylation with gas chromatographic quantification. (b) Gal content as determined by *per*‐acetylation with gas chromatographic quantification. (c) Ratio of Ara: Gal. The lollipop plot shows the Gal content relative to the Ara content (Ara = 1). (d) Hyp content (in % w/w) as determined colourimetrically. (e) Illustration of the connection of Hyp, Ara and Gal in arabinogalactan‐proteins (AGPs). All results are given as mean values (*n* = 3). [Color figure can be viewed at wileyonlinelibrary.com]

The absolute amounts of Ara and Gal were calculated (Figure 4a,b, Supporting Information: Table S1a–c) and found to be highest in roots compared to leaves and lowest in old leaves. Under low and adequate boron supplementation, salt‐grown plants always had lower amounts of Ara and Gal.

The ratio of Ara: Gal is lower in roots (= more Gal) and comparable higher in old and young leaves (Figure 4c). In all organs, salt‐stressed plants were characterised by a slightly higher Ara: Gal ratio (= more Ara). No influence of boron on the Ara: Gal ratio could be detected.

As Hyp is responsible for binding of AG‐glycans to the protein moiety in AGPs, the content of Hyp was additionally determined (Figure 4d). Comparable to results for Ara and Gal, roots contained the highest Hyp amounts, and the influence of growth conditions was also comparable. To conclude, in LB and AB, salt‐grown plants always contained less Ara, Gal and Hyp. There was no influence of boron on Ara, Gal and Hyp content.

#### 3.5.3 Radial Diffusion Assay

**Figure 5:**
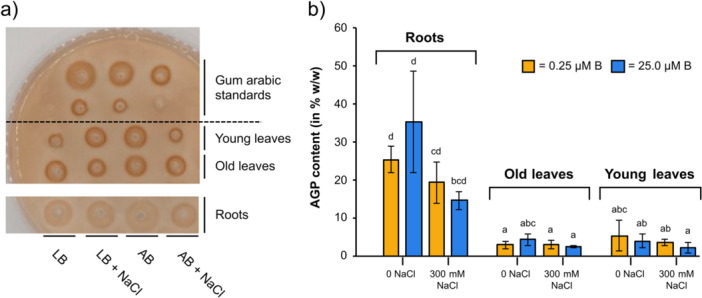
Amounts of AGPs in roots and leaves of sugar beet under different salt (0 or 300 mM NaCl) and boron (LB: low boron, 0.25 µM B; AB: adequate boron, 25.0 µM B) conditions, determined by radial gel diffusion with Yariv′s reagent. (a) One replicate of the agarose gel loaded with standards and samples. (b) AGP content determined by standard regression with gum arabic. [Color figure can be viewed at wileyonlinelibrary.com]

A typical feature of AGPs is interaction with the so‐called Yariv's reagent. Specific precipitation of AGPs with this red‐coloured dye offers the possibility of quantification of AGPs in a radial gel diffusion assay (Figure 5). Different concentrations of gum arabic were used as standard AGP samples for calibration (Figure 5a). As expected from compositional analyses, amounts of AGPs were highest in roots (15.6%–25.2% AE) and much lower in leaves (old leaves: 2.3%–4.3% in AE; young leaves: 2.2%–5.3% in AE; Figure 5b). Although statistically not significant, there was a trend that salt stress led to reduced AGP content in all organs and could not be compensated by adequate boron supply. In roots and old leaves, the highest AGP amounts were found with adequate boron without salt stress.

Our results show that the tested growth conditions have a statistically relatively low influence on AGP content in roots and leaves. Boron is not affecting AGP content or composition, whereas salt‐stress leads to reduced levels of AGPs with slightly increased ratios of Ara: Gal.

### 3.6 Influence of Salt and Boron on Pectic Polysaccharides

#### 3.6.1 Yield

Yields of the two pectic fractions were between 2.5%–5.5% (AmOx fraction) and 2.9%–7.4% (Na_2_CO_3_ fraction) and higher in leaves compared to roots. With regard to salt and boron conditions, no statistically relevant differences were detected (Supporting Information: Figures S8 and S10).

#### 3.6.2 Composition

**Figure 6:**
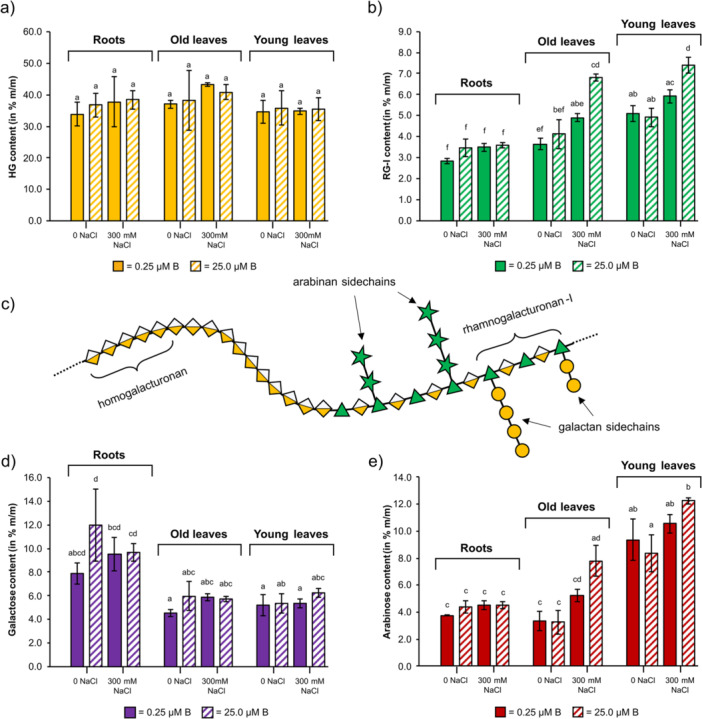
Changes in pectic components under shifting boron and salt conditions as inferred from silylation analysis of the ammonium oxalate fractions. (a) Homogalacturonan (HG) contents. (b) Content of rhamnogalacturonan‐I (RG‐I) core backbone structures. (c) Schematic structure of pectic macromolecule containing HG and RG‐I backbones, including galactan and arabinan sidechains. (d, e) Content of RG‐I sidechain structures (d: galactose content, e: arabinose content). All values were inferred from silylation analysis performed in triplicate (*n* = 3). [Color figure can be viewed at wileyonlinelibrary.com]

The monosaccharide composition of all pectic fractions is shown in Supporting Information: Figures S9, S11 and Table S2,3. Ara, Gal and Glc were the dominating neutral monosaccharides of all organs under all conditions. Furthermore, Rha was always present in appreciable amounts. Ara, Gal and Rha are the dominating neutral monosaccharides in pectins, whereas Glc might originate from starch. Other neutral monosaccharides present are Xyl, Man and sometimes Fuc. A difference between the both fractions is that all Na_2_CO_3_ fractions are richer in Ara. Man and Xyl are more abundant in roots in both fractions. Silylation analyses of the pectic AmOx fraction enabled determination of GalA also in relation to the neutral monosaccharides (Figure 6). The content of HG, RG‐I (for structures, see Figure 6c) and Ara and Gal as sidechains was inferred from the results. While HG contents were very similar between the different treatments (Figure 6a), RG‐I contents in leaves were significantly enriched under adequate boron and high salt conditions (Figure 6b). There was a general trend detectable with higher Gal in roots (Figure 6d) and higher Ara in leaves (especially young leaves; Figure 6e). Under adequate boron conditions, the salt addition significantly elevated the Ara contents in leaves (Figure 6e).

To detect extensins, we determined the Hyp content in all Na_2_CO_3_ extracts (Supporting Information: Figure S12). Hyp content was higher in roots compared to leaves, but the different growth conditions had no influence on Hyp amounts in these pectic extracts.

#### 3.6.3 PCA

Monosaccharide composition of all fractions was analysed by PCA (Supporting Information: Figure S13) and differed between the AEs and the two pectic fractions, which partly overlapped (Supporting Information: Figure S13a). Galactose has the biggest impact on the water‐soluble fractions, whereas influence of Rha is bigger on the pectic fractions. The different growth conditions showed great similarities, most prominent in the two conditions 0.25 B + salt tress and 25 B + salt stress (Supporting Information: Figure S13b). Inclusion of boron and salt concentrations revealed differences in the water‐soluble and sodium carbonate fractions, whereas AmOx fractions partly overlapped with the Na_2_CO_3_ fractions and also with the AEs. A close connection of salt and Ara is noticeable (Supporting Information: Figure S13c). This becomes even more clear in Supporting Information: Figure S13d. There is a stacking of the fractions from 0.25 B without salt at the top, followed by 25 B without salt, 0.25 B + salt stress and finally 25 B + salt stress at the bottom with clear correlation of Ara and salt.

#### 3.6.4 RG‐II: PAGE

**Figure 7:**
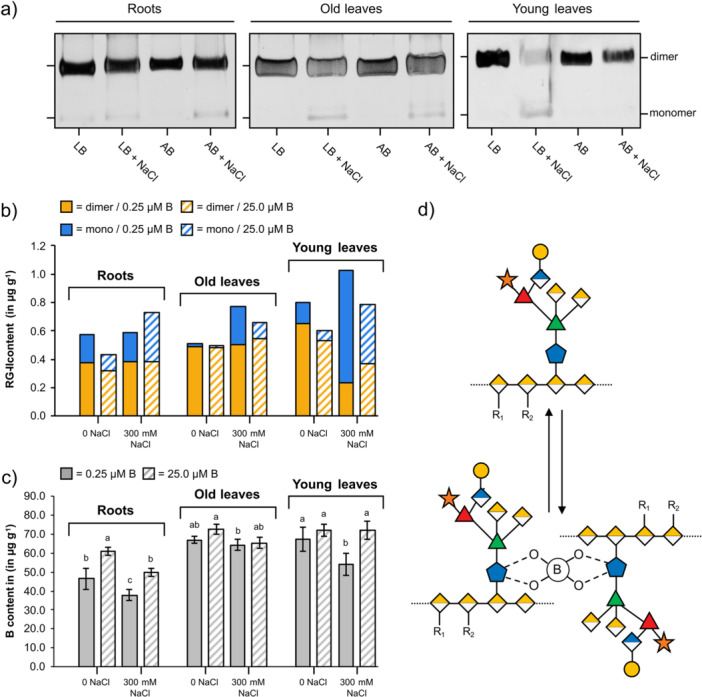
Interconnection of rhamnogalacturonan‐II (RG‐II) and boron contents (a) Polyacrylamide gel electrophoresis with polysaccharide fractions released by EPG‐digestion from roots and leaves of sugar beet under different salt (0 or 300 mM NaCl) and boron (LB: low boron, 0.25 µM B; AB: adequate boron, 25.0 µM B) conditions. (b) Quantification of RG‐II monomer and dimer in polysaccharide fractions from roots and leaves of sugar beet under different salt and boron conditions (*n* = 3, for details see Figure S14) (c) Boron content in ammonium oxalate fraction determined by ICP‐MS measurements (*n* = 3). (d) Schematic representation of the transfer from monomer to dimer state of RG‐II. Connection is formed by the covalent binding of two apiose residues to boron. [Color figure can be viewed at wileyonlinelibrary.com]

Cross‐linking of the pectin domain RG‐II via boron bridges between two apiose residues (Figure 7d) is necessary for a functional cell wall. PAGE enabled us to detect the RG‐II monomer and dimer (Sanhueza et al. 2022). Figure 7a shows exemplary gels for all samples with general dominance of the RG‐II dimer in nearly all conditions. Only in the young leaves of LB salt‐stressed plants, more monomer than dimer was present.

##### 3.6.4.1 Influence of Salt

Quantification of dimer and monomer bands (Figure 7b) revealed a higher monomer content in leaves and roots of salt‐stressed plants under LB and AB conditions. The increase of the monomer under salt stress was most prominent in young leaves, followed by old leaves and finally roots.

##### 3.6.4.2 Influence of Boron

Quantification of B in the AmOx fractions of all organs (Figure 7c) revealed slightly lower amounts in roots. This is in accordance with lower amounts of dimer in roots in salt‐free conditions. In all organs of plants grown without salt, the AB condition led to slightly higher amounts of cross‐linked dimers. In roots, AB did not result in a higher dimer amount compared to LB under salt stress. In contrast, in young and old leaves, AB was able to partially heal the influence of salt: under salt stress, the amount of dimer increased when adequate boron was offered.

## 4 Discussion

Plant cell walls are dynamic and complex structures, acting as a first line of defence against environmental stressors such as salinity. Land cultivated plants are significantly affected by salinity and nutrient imbalances that are interrelated in determining the architecture and function of the cell wall. Boron is one of these nutrients, and a saline environment reduces the water solubility of boron, causing a deficiency. However, the physical interaction between salinity and boron remains less documented.

### 4.1 Adequate Boron Mitigates Salt‐Induced Growth Inhibition

Sufficient availability of boron enhances salinity resistance in sugar beet plants under high salinity stress conditions. Results showed that both fresh and dry matter were significantly higher with an adequate supply of boron (25 μM) than with a low boron (0.25 μM) supply in 300 mM NaCl‐grown sugar beet plants (Figure 1a,b, Supporting Information: S4a,b). Nicolas‐Espinosa et al. (2024) observed similar changes for broccoli under a combination of salt and boron deficiency. However, at the subcellular level, the combined effect of high boron and salt antagonistically affects wheat growth (Masood et al. 2012). In contrast, the present investigation has elucidated that salinity resistance is enhanced in boron‐demanding dicot sugar beet and exhibits more living cells at AB as compared to the LB plant in both short and long‐time salt stress conditions (Figure 1a,c, Supporting Information: Figure S2a–d), supporting this notion for barley, wheat, cotton and also sugar beet seedlings (Wimmer and Goldbach 2012; Dong et al. 2021; Lu et al. 2023; Qu, Havshøi, et al. 2024).

Salinity adaptation is related to leaf growth (Munns and Tester 2008; Pitann et al. 2011). The results in this study indicated a significant enlargement of the young leaf tissue area and a reduced leaf apoplastic pH, which confers acid growth by AB‐treated sugar beet under higher salinity (Figure 1c–e). The increase in leaf tissue area is important for diluting excessive salt and supports the observation of cell extension in *Atriplex hastata* (Black 1958). The acidification of the leaf apoplast enhances the salinity resistance of *Beta vulgaris* and genotypes of *Zea mays*. Consequently, it promotes acid growth (Pitann et al. 2009; Wakeel et al. 2010). Furthermore, photosynthetic ability and Rubisco (ribulose‐1,5‐bisphosphate carboxylase/oxygenase) activities of sugar beet positively correlate with salinity resistance (Hossain et al. 2017). Boron is imperative for photosynthesis in sugar beet (Song et al. 2023) and the overall vitality of plants because deficiency inhibits the activities of Rubisco as found in citrus leaves (Han et al. 2008). Additionally, drought and boron deficiency intensify membrane damage in sugar beet leaves, disrupting source‐sink relations by inhibiting photosynthetic assimilates (Chowdhury et al. 2025). A sufficient supply of boron enhances carbohydrate synthesis, for example, sucrose, in sugarcane and tobacco leaves (Shi et al. 2012; Martello et al. 2025). Increasing osmolytes improves the water retention capacity of the stressed cells. The mobility and cellular osmotic potential of sucrose further act as a driving force for water to move from the xylem to phloem (Chiang et al. 2013; Yousefi et al. 2020). Water uptake modifies the boron uptake mechanism. Boron translocation through the xylem is driven by the water potential gradient generated *via* leaf transpiration. In passive diffusion, boron absorption by the root cells and its subsequent transport through the xylem to the shoot depend on the transpiration stream and functionally on aquaporins (Miwa and Fujiwara 2010; for review, see Wimmer and Eichert 2013). Oxidative stress, for example, salinity, drought and boron deficiency, interferes with water transport by altering the structure of xylem vessels and the osmolyte accumulation patterns. Therefore, the downregulation of aquaporins and reduced leaf transpiration caused by NaCl stress indicate stress conditions (Gao et al. 2002; Martinez‐Ballesta et al. 2008). In dicot plants, inhibition of root growth further decreases hydraulic conductivity, leading to stomatal closure and reducing water flow driven by transpiration (Hu and Brown 1997; Wimmer and Eichert 2013). The studies have reported that a sufficient boron supply increases root growth under both normal and stressful conditions (Chowdhury et al. 2024, 2025; Qu, Havshøi, et al. 2024; Lu et al. 2023; 2025). This enhances transpiration‐driven boron translocation *via* the upregulation of aquaporins (Nicolas‐Espinosa et al. 2024). In line with earlier findings, this investigation observed that a higher transpired volume of water was observed at the AB supply under high salinity conditions during final harvesting (Figure 2a), indicating the need to explore the influence of water relations on salinity stress resistance. At the inception of the treatment period, AB plants exhibited an increased transpiration rate (*Tr*) under higher salinity stress. After passing the first phase of salt stress, the rate was decreased (middle), still reaching levels higher than in LB‐treated plants (Figure 2b). In contrast to LB, in nonsaline conditions, no decline was seen in AB‐treated plants throughout the study. This attribute indicates that plants' boron levels did not reach toxicity in their leaves (Figure 1a,c, Supporting Information: S2a,b) and sugar beet plants tried to utilise boron under saline conditions (Figure 2b). Furthermore, after the first phase of salt stress, stomatal conductance also shows comparable results between salt‐grown LB and AB plants (Figure 2c). The differences remained visible until harvesting of LB plants (Figure 2c), which was well documented by Kronzucker et al. (2013). Thus, this confirms the retrieval of disturbed photosynthesis machinery in the presence of boron. In this study, salinity impacts photosynthesis, and a decline in photosynthesis rate was observed (Figure 2d). LB‐treated plants exaggerate such a condition, and analogous chlorophyll changes were shown similarly by SPAD value (Figure 2e). However, AB‐treated plants enhance photosynthesis like stomatal conductance without or with salt‐grown conditions after the first phase until harvesting (Figure 2c,d). Reduced stomatal conductance and transpiration under NaCl stress in LB‐treated plants corresponded to boron toxicity conditions in *Arabidopsis*. Boron toxicity inhibits growth and down regulates aquaporins by increasing the expression of abscisic acid‐related genes, thereby reducing stomatal function (Macho‐Rivero et al. 2017). In contrast, the supply of soil boron reduces cellular damage in *Pistacia vera* seedlings under NaCl stress by enhancing the production of osmolytes, such as glycine betaine, which increases water availability (Karimi et al. 2018). This phenomenon indicates an osmotic adjustment and better salt tolerance with higher photosynthetic performance (Hossain et al. 2017).

### 4.2 Boron Regulates Ion Balance in Sugar Beet Plants Subjected to Salt Stress

As the process of photosynthesis in leaves undergoes enhancement following the provision of boron under conditions of salt stress, a query emerges concerning the correlation between the observed alterations in the presence of boron under saline conditions and the changes in ionic relations within the tissue and subcellular sap fluid of sink leaves. Therefore, the apoplastic and symplastic fluids from young leaves were isolated and analysed. It was observed that boron (^10^B) uptake was affected in both fluids from young leaves (Figure 1g). Also, the dry matter of the root under high salt stress, but not in the dry matter of the leaves of AB‐treated plants (Figure 1f). The concentrations of boron (^10^B) in both apoplastic and symplastic fluids were considerably elevated in AB salt‐grown plants compared to LB‐grown plants, irrespective of salinity (Figure 1g). Similar results were observed for the boron (^10^B) concentrations in the shoots and roots of the short‐time salinity experiment (Supporting Information: Figure S2a,b). In the long‐time salinity experiment, it was also obtained that boron (^10^B) accumulation (Supporting Information: Figure S4c,d) and growth of leaves and roots were synergistically increased in AB‐treated sugar beet plants under higher salinity (Figure 1a–d). These results depicted that AB supply under high salinity, transpiration and leaf area is a dominant factor in the accumulation and translocation of boron in the leaves compared to LB (Figures 1, 2). Via passive uptake, transpiration is a decisive factor at high boron supply rather than active boron uptake through diffusion at limited supply (Yermiyahu et al. 2008; Wimmer and Goldbach 2012). The results show that the growth reduction of sugar beet due to salinity depends on the supplied boron and salinity levels (Supporting Information: Figure S3a–d). Moreover, results revealed that salinity increases Na^+^ concentrations in the shoot (Supporting Information: Figure S3c,d), mainly in old leaves compared to young leaves and roots (Figure 3a). Comparing boron levels, this concentration was significantly higher in the LB‐treated plants in all parts, compared to the AB treatment (Figure 3a, Supporting Information: S3a,b). However, no significant changes were observed in either leaf fluids (Figure 3b). The exclusion of Na^+^ from leaves and roots for salinity resistance in plants is closely related to the declining ratio of Na^+^/K^+^ and Na^+^/Ca^2+^ (Weng et al. 2022; Dissanayake et al. 2024), as salinity negatively impacts the transpiration stream, affects growth (Figures 1a–c, 2a,b) and the uptake of K^+^ and Ca^2+^ (Supporting Information: Figure S5a–d). Aline with our AB‐treated sink leaves growth (Figure 1c‐d), prior experiments indicated that the exclusion of Na^+^ from leaf blades is a strategy to tolerate higher salinity levels (Munns and Tester 2008). It is a known fact that salinity reduces the movement of K^+^, and an excessive amount of positively charged Na^+^ at the plant cell wall displaces Ca^2+^ at the cell wall binding site (Steinhorst et al. 2022; Shomer et al. 2003; O'Neill et al. 2004; Byrt et al. 2018). This is mainly due to Na^+^ influx via K^+^ pathways (Blumwald 2000), and reduced Ca^2+^ function in the cell wall structure and membrane integrity to tolerate ionic stress (Proseus and Boyer 2012). This ultimately affects the growth and yield of the plant (Munns and Tester 2008; Zhu et al. 2017). Our investigation revealed that LB significantly reduces K^+^ and Ca^2+^ concentrations (Supporting Information: Figure S5a–d), and higher salinity increases the Na^+^/K^+^ ratio in the tissue and the leaf fluids (Figure 3c,d, Supporting Information: Figure S3e–h), compared to the AB‐treated sugar beet plant. Optimising K^+^/Na^+^ homoeostasis in response to excessive salt is crucial for plant salt resistance and halotropism (Qu, Havshøi, et al. 2024; Hua et al. 2024). In addition, higher levels of boron positively enhance Ca^2+^ transport (Läuchli 2007; Bastías et al. 2010) and stabilise pectic polysaccharides in the cell wall (Shi et al. 2017). The results depicted that Na^+^/Ca^2+^ ratios considerably explain this observation (Figure 3e–f). Findings revealed that under higher salinity in LB‐treated plants, the ratio of Na^+^/Ca^2+^ was higher in all tissues and fluids than in AB‐treated plants. Shabala and Cuin (2008) suggested that Ca^2+^ plays an integral role in lowering Na^+^ toxicity, for example, by increasing the uptake of K^+^ and facilitating the amelioration of salt damage. It has also been reported that salt stress causes Ca^2+^ deficiency in plants and disturbs the balance of the K^+^/Na^+^ in membranes (Zhu et al. 2017). Further, to understand the influence of boron in cell wall dynamics regarding interference of excessive Na^+^, highly glycosylated AGPs, moderately glycosylated EXTs and cell wall remodelling of RG‐II were investigated.

### 4.3 Influence of B and Salt‐Stress on Cell Wall AGPs

Analysis of neutral monosaccharide composition and radial gel diffusion revealed the presence of AGPs in young and old leaves as well as in roots of *Beta vulgaris*. The content in roots was more than five times higher compared to leaves. Another investigation on sunflower, peanut and cotton detected 5 to 10 times higher concentrations of Hyp in roots compared to stems and leaves, possibly due to higher AGP and maybe also extensin amounts in roots (Golan‐Goldhirsh et al. 1990).

With regard to growth conditions, the results of this study reveal only a low impact on AGP content and composition. No effect of boron could be detected. Salt‐stress led to slightly reduced amounts of Ara, Gal and Hyp, thus reflecting reduced levels of AGPs. In sunflower, peanut and cotton, the effect of salt treatment on Hyp concentrations in stems, leaves and roots was not significant (Golan‐Goldhirsh et al. 1990). The role of AGPs during salt stress seems to be multifaceted, from decrease with a stiffening effect on cell walls to increase of extracellular AGPs to activate signalling mechanisms (for review, see Mareri et al. 2019). In secondary walls of xylem elements of *Brassica oleracea*, AGP abundance was reduced under salt stress, probably to reduce plasticity and support xylem stiffness (Fernandez‐Garcia et al. 2011). In liquid cell cultures with salt, the amount of AGPs in the growth media increased strongly (Lamport et al. 2006; Zagorchev et al. 2008), whereas there was no significant difference in the cell wall fraction (Zagorchev et al. 2008). Plants with defects in production of AGPs are hypersensitive to high salinity (for review, see Zhao et al. 2020).

The Ara: Gal ratio in roots was lower compared to leaves, indicating either a different structure of AGPs or the presence of arabinans besides AGPs, especially in leaves. Salt stress induced a slight increase in the Ara: Gal content in roots and leaves, which might also be due to a change in AGP structure or an increase in arabinan not bound to AGP (e.g., sidechains of coextracted RG‐I). Furthermore, PCA of monosaccharide composition hinted at a correlation between salt stress and Ara (Supporting Information: Figure S13c,d). Arabinans have been described to be involved in salt stress tolerance in *Artemisia annua* (Corrȇa‐Ferreira et al. 2019), and the importance of Ara metabolism in salt stress tolerance was also shown for *Arabidopsis* (Zhao et al. 2019). Mutations in the *MUR4* gene, which is needed for the biosynthesis of UDP‐Ara*p* in *Arabidopsis*, led to reduced root elongation during salt stress, and this short root phenotype could be rescued by exogenous Ara (Zhao et al. 2019). Furthermore, arabinosylation of extensins is an important response to salinity in roots of *Arabidopsis* (Zou et al. 2024). To search for extensins, we determined the Hyp content in the Na_2_CO_3_ fractions. Hyp and possible extensins were present in leaves and roots under all growth conditions, but no influence of salt or boron could be shown (Supporting Information: Figure S12).

### 4.4 Influence of B and Salt Stress on Cell Wall Pectins

The structure of pectins from *Beta vulgaris* has been investigated in detail (Strasser 2001 and 2002). The RG‐I backbone consisting of alternating GalA and Rha is highly ramified, as the Rha residues are highly branched. The side chains consist of arabinans, galactans and arabinogalactans, thus reflecting a typical composition of an angiosperm RG‐I (Strasser 2001). The amount of RG‐II in dicotyledons and nongraminaceous monocotyledons was estimated to be in mean 22.8 mg/g of the dry cell wall (*n* = 18), which was substantially higher compared to the Gramineae and spore‐producing land plants (Matsunaga et al. 2004). RG‐II structure is highly conserved (O'Neill et al. 2004) and also present in *Beta vulgaris* with slight variations (Ishii and Matsunaga 1996). In primary walls, two RG‐II monomers are cross‐linked by a borate diester *via* apiose to form an RG‐II dimer, which was first shown by NMR of in vitro RG‐II borate complexes derived from *Beta vulgaris* (Ishii and Matsunaga 1996). This covalent cross‐linking of RG‐II together with Ca^2+^ dependent egg‐box formation of HG (Grant et al. 1973) is necessary for the formation of a three‐dimensional pectin network in the plant cell wall.

In this investigation, no statistically relevant differences were detected in the yields or the neutral monosaccharide composition of the two pectic fractions with regard to the different salt and boron conditions, but PCA revealed a correlation of salt stress and Ara (Supporting Information: Figure S13c,d). The above‐mentioned increase in RG‐I‐bound arabinan and HG demethylation in *Artemisia annua* under salinity stress (Corrêa‐Ferreira et al. 2019) corresponds to that finding. While we did not focus on degree of methylation in pectins, the former applies to our data. Similar to the study of Liu et al. (2025) on spinach roots, another member of the Amaranthaceae family, we saw an overall constant content of Ara under different conditions. As our study also included leave data, an increase of Ara under salinity stress was detectable (Figure 6e). We therefore hypothesise varying consequences on pectin structures in the different organs.

Further, an investigation revealed that salt stress increased the amount of cell wall pectins, and boron supply under salt stress reduced the yields of chelated and alkali‐soluble pectins (Lu et al. 2023). Our yield data (Supporting Information: Figure S8) did not allow this conclusion, but silylation data hint towards a reorganisation of pectin structure during that process (Figure 6e). While HG contents remained more or less similar between treatments (Figure 6a), RG‐I contents increased under salinity—significantly under adequate boron treatment (Figure 6b).

Furthermore, we found an influence on RG‐II cross‐linking dependent on the growth conditions (Figure 7b,c). Under salt stress, cross‐linking of RG‐II was decreased and the amount of monomer increased. This means that salinity disrupts pectin cross‐linking, leading to softening of the cell wall (Qu, Huang, et al. 2024). It is known that Ca^2+^ promotes cross‐linking, and excess Na^+^ may displace Ca^2+^, thereby hindering dimerisation (Byrt et al. 2018). This effect was much more pronounced in leaves compared to roots and strongest in young leaves (Figure 3e). The higher Na^+^/Ca^2+^ ratio was detected in young leaves and apoplastic fluids due to the influence of low boron (0.25 μM) (Figure 3e–f). This was further confirmed by lower boron concentration in the ammonium oxalate fractions (Figure 7c). In the sunflower plant, low boron causes swelling of the cell walls and a larger pore size, indicating free boron binding sites in the cell wall (Dannel and Pfeffer 2000; see review Dannel et al. 2002). Therefore, reduced cross‐linking (Fleischer et al. 1999) and decreased RG‐II dimerisation leads to a lack of coordination between growth and cell adhesion (Baba et al. 2024). Furthermore, sugar beet plants under salt stress allow higher Na^+^ (Yang et al. 2012; Hossain et al. 2017). Boron predominantly accumulates in the leaf rather than in the root (Guidong et al. 2011), and deficiency results in reduced water transport to the aerial parts of the plant (Wimmer and Eichert 2013). Consequently, this leads to limited translocation, particularly exacerbated under saline conditions (Figures 1f–g, 2a,b). Recently, we found that adequate boron in *Beta vulgaris* offers higher phloem mobility from old to young leaves, which confers sink strength under drought stress (Chowdhury et al. 2025). In rapeseed, boron confers declining translocation of Na^+^ from root to shoot, which is facilitated by *BnaA2. HKT1* (Hua et al. 2024). Our results also indicated that without salt stress, adequate boron supply led to a slightly higher RG‐II dimer content in roots and leaves (Figure 7b). This is in correspondence to earlier findings that the proportion of RG‐II dimer increased with boron supply (Matsunaga and Ishii 2006). In young and old leaves, adequate boron alleviated salt stress and the amount of dimer increased in comparison to the low boron conditions. In general, adequate boron supply improves the potential of plants to cope with salt stress (Qu, Huang, et al. 2024), and our results underline that part of this beneficial role is due to support of RG‐II dimer formation. However, as the bioavailability of boron and Na^+^ differs between hydroponic and field conditions, the stability of RG‐II dimerisation may change over time and vary among cultivars. To generalise the results, future studies should be worked on our limitations, which include (1) environmental applicability, (2) long‐term assessment and (3) cultivar specificity. Although we detected no effect of boron on AGP content, it is important to mention that these glycoproteins catalyse the boron bridging of RG‐II (Sanhueza et al. 2022).

## 5 Conclusions

Halophytic sugar beet plants have been shown to possess the capacity to thrive in highly saline environments (Yang et al. 2012). However, accumulation of Na^+^ has been demonstrated to physically interfere with the cell wall structure. In the present study, our novel findings propose a correlation between the salt resistance of sugar beet and the structural modification of the cell wall. In this regard, it has been demonstrated that boron promotes growth and enhances salinity resilience of dicot plants. Moreover, an adequate presence of boron counteracts Ca^2+^ displacement by Na^+^, particularly from sink leaves. Our findings indicate an increase in RG‐I in leaves under adequate boron and high salt conditions, as well as an increased translocation of boron from old to young leaves and higher amounts of dimeric RG‐II, which confers the strength of the sink cell wall against salt stress.

## Conflicts of Interest

The authors declare no conflicts of interest.

## Supporting information


**Figure S1:** A schematic illustration of the experimental setup, different samples, and their respective uses after harvesting. **Figure S2:** Boron (^10^B) concentration in the shoot (a), root (b), phenotypic overview at 24 hours (hr) (c) and 48 hours (d) of sugar beet plants under different ^10^B treatment conditions with 0 and 300 mM NaCl salt stress. **Figure S3:** 1 Root, shoot dry matter (a, b), concentrations of Na^+^ (c, d), the ratio of Na^+^/K^+^ (e, f), Na^+^/Ca^2+^ (g, h) in the root and shoot under different levels of salt‐grown sugar beet plants at different boron conditions. **Figure S4:** 4 Root, shoot fresh matter (a, b), accumulation of boron (^10^B) (c, d) in the root, and shoot of high salt‐grown sugar beet plants at low and adequate boron treatment. **Figure S5:** Quantification of K^+^ (a, b), and Ca^2+^ (c, d) concentrations in the roots, young and old leaves, leaf apoplastic and symplastic fluids of sugar beet plants under low (0.25 μM ^10^B; LB) and adequate (25 μM ^10^B; AB) levels of boron after NaCl stress (0 mM NaCl/300 mM NaCl). Sugar beet plants were cultivated in Hoagland's nutrient solution for 28 days at LB and AB conditions. **Figure S6:** Yields of water‐soluble fractions from the roots and leaves of *Beta vulgaris* under different salt and boron conditions. **Figure S7:** Neutral monosaccharide composition of water‐soluble fractions from the roots and leaves of *Beta vulgaris* under different salt and boron conditions. **Figure S8:** Yields of the pectic oxalate fraction from the roots and leaves of *Beta vulgaris* under different salt and boron conditions. **Figure S9:** Neutral monosaccharide composition of the pectic oxalate fraction from the roots and leaves of *Beta vulgaris* under different salt and boron conditions. **Figure S10:** Yields of pectic Na_2_CO_3_ fractions from the roots and leaves of *Beta vulgaris* under different salt and boron conditions. **Figure S11:** Neutral monosaccharide composition of pectic Na_2_CO_3_ fractions from the roots and leaves of *Beta vulgaris* under different salt and boron conditions. **Figure S12:** Amounts of Hyp in Na_2_CO_3_ fractions from the roots and leaves of *Beta vulgaris* under different salt and boron conditions. **Figure S13:** Principal component analysis of monosaccharide composition of the different cell wall fractions (aqueous extract, ammonium oxalate, sodium carbonate). **Figure S14:** Contents of RG‐II monomers and dimers in roots, old leaves and young leaves of *Beta vulgaris* grown under different treatments. **Table S1a:** Neutral monosaccharide composition of the aqueous fractions from **roots** of *Beta vulgaris* grown under different salt and boron conditions in % (mol mol^−1^; *n* = 3; tr: trace value < 1 %). **Table S1b:** Neutral monosaccharide composition of the aqueous fractions from **old leaves** of *Beta vulgaris* grown under different salt and boron conditions in % (mol mol^−1^; *n* = 3; tr: trace value < 1 %). **Table S1c:** Neutral monosaccharide composition of the aqueous fractions from **young leaves** of *Beta vulgaris* grown under different salt and boron conditions in % (mol mol^−1^; *n* = 3; tr: trace value < 1 %). **Table S2a:** Neutral monosaccharide composition of the ammonium oxalate fractions from roots of *Beta vulgaris* grown under different salt and boron conditions in % (mol mol^−1^; *n* = 3; tr: trace value < 1 %). **Table S2b:** Neutral monosaccharide composition of the ammonium oxalate fractions from old leaves of *Beta vulgaris* grown under different salt and boron conditions in % (mol mol^−1^; *n* = 3; tr: trace value < 1 %). **Table S2c:** Neutral monosaccharide composition of the ammonium oxalate fractions from young leaves of *Beta vulgaris* grown under different salt and boron conditions in % (mol mol^−1^; *n* = 3; tr: trace value < 1 %). **Table S3a:** Neutral monosaccharide composition of the sodium carbonate fractions from **roots** of *Beta vulgaris* grown under different salt and boron conditions in % (mol mol^−1^; *n* = 3; tr: trace value < 1 %). **Table S3b:** Neutral monosaccharide composition of the sodium carbonate fractions from **old leaves** of *Beta vulgaris* grown under different salt and boron conditions in % (mol mol^−1^; *n* = 3; tr: trace value < 1 %). **Table S3c:** Neutral monosaccharide composition of the sodium carbonate fractions from **young leaves** of *Beta vulgaris* grown under different salt and boron conditions in % (mol mol^−1^; *n* = 3; tr: trace value < 1 %).

## Data Availability

The data that support the findings of this study are available from the corresponding author upon reasonable request. Relevant to the findings, all experimental data can be obtained within the manuscript and supporting information.
