# Supplementary material for: Rhamnogalacturonan‐II Dimerisation Reinforces Salt Resistance in Sugar Beet
Source: Plant Cell Environ. 2026 Feb 19;49(6):3082–100. doi: 10.1111/pce.70457 (PMC13136558; doi:10.1111/pce.70457)
Supplement: Supplementary file 1 — Figure S1: A schematic illustration of the experimental setup, different samples, and their respective uses after harvesting. Figure S2: Boron (10B) concentration in the shoot (a), root (b), phenotypic overview at 24 hours (hr) (c) and 48 hours (d) of sugar beet plants under different 10B treatment conditions with 0 and 300 mM NaCl salt stress. Figure S3: 1 Root, shoot dry matter (a, b), concentrations of Na+ (c, d), the ratio of Na+/K+ (e, f), Na+/Ca2+ (g, h) in the root and shoot under different levels of salt‐grown sugar beet plants at different boron conditions. Figure S4: 4 Root, shoot fresh matter (a, b), accumulation of boron (10B) (c, d) in the root, and shoot of high salt‐grown sugar beet plants at low and adequate boron treatment. Figure S5: Quantification of K+ (a, b), and Ca2+ (c, d) concentrations in the roots, young and old leaves, leaf apoplastic and symplastic fluids of sugar beet plants under low (0.25 μM 10B; LB) and adequate (25 μM 10B; AB) levels of boron after NaCl stress (0 mM NaCl/300 mM NaCl). Sugar beet plants were cultivated in Hoagland's nutrient solution for 28 days at LB and AB conditions. Figure S6: Yields of water‐soluble fractions from the roots and leaves of Beta vulgaris under different salt and boron conditions. Figure S7: Neutral monosaccharide composition of water‐soluble fractions from the roots and leaves of Beta vulgaris under different salt and boron conditions. Figure S8: Yields of the pectic oxalate fraction from the roots and leaves of Beta vulgaris under different salt and boron conditions. Figure S9: Neutral monosaccharide composition of the pectic oxalate fraction from the roots and leaves of Beta vulgaris under different salt and boron conditions. Figure S10: Yields of pectic Na2CO3 fractions from the roots and leaves of Beta vulgaris under different salt and boron conditions. Figure S11: Neutral monosaccharide composition of pectic Na2CO3 fractions from the roots and leaves of Beta vulgaris under different salt and bo [file PCE-49-3082-s001.pdf]

## Supplementary data to:

# Rhamnogalacturonan-II dimerization reinforces salt resistance in sugar beet

Md. Shah Newaz Chowdhury[1], Lukas Pfeifer[2], Kim-Kristine Mueller[2], Md. Sazzad Hossain[1], Birgit Classen[2]*, and Karl Hermann Mühling[1]*

[1]*Institute of Plant Nutrition and Soil Science, Kiel University, Hermann-Rodewaldstrasse 2, 24118 Kiel, Germany*

[2]*Pharmaceutical Institute, Department of Pharmaceutical Biology, Kiel University, Gutenbergstrasse 76, 24118 Kiel, Germany*

***Corresponding authors**:

Birgit Classen, Pharmaceutical Institute, Department of Pharmaceutical Biology, Kiel University, *Gutenbergstrasse 76*24118 Kiel, Germany

E-mail: bclassen@pharmazie.uni-kiel.de; Phone: +49-431-8801130

Karl Hermann Mühling, Institute of Plant Nutrition and Soil Science, Kiel University, Hermann-Rodewald-Str. 2, 24118 Kiel, Germany.

E-mail: khmuehling@plantnutrition.uni-kiel.de; Phone: +49-431-8803189

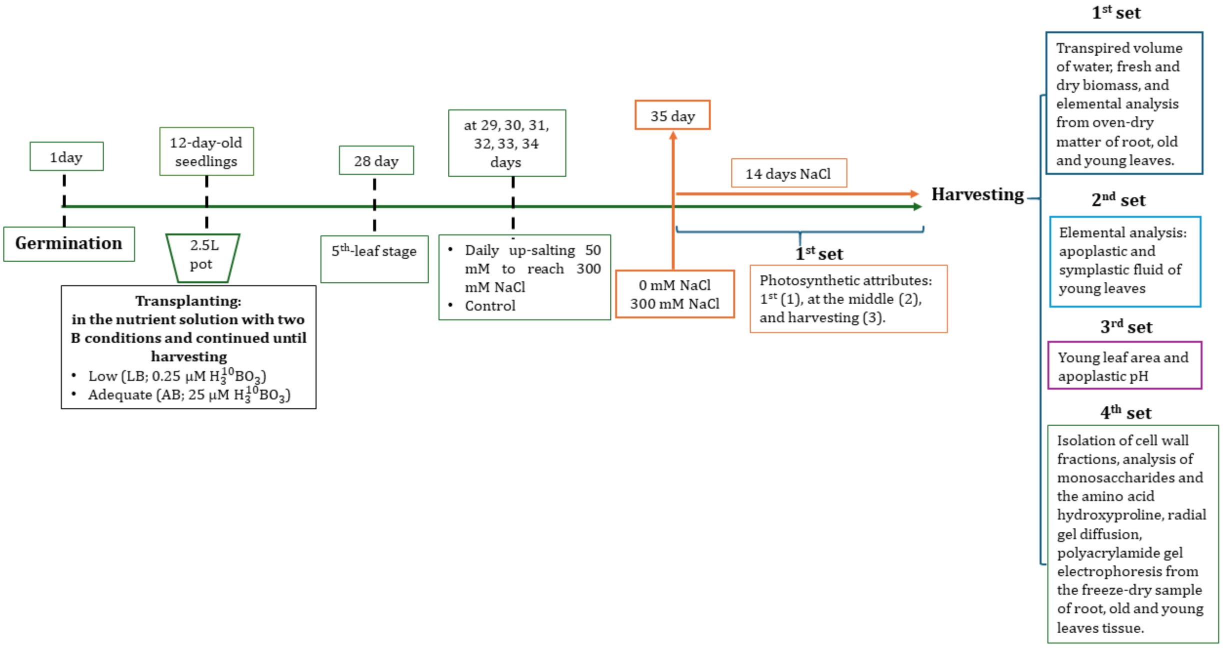


**Figure S1.** A schematic illustration of the experimental setup, different samples, and their respective uses after harvesting.

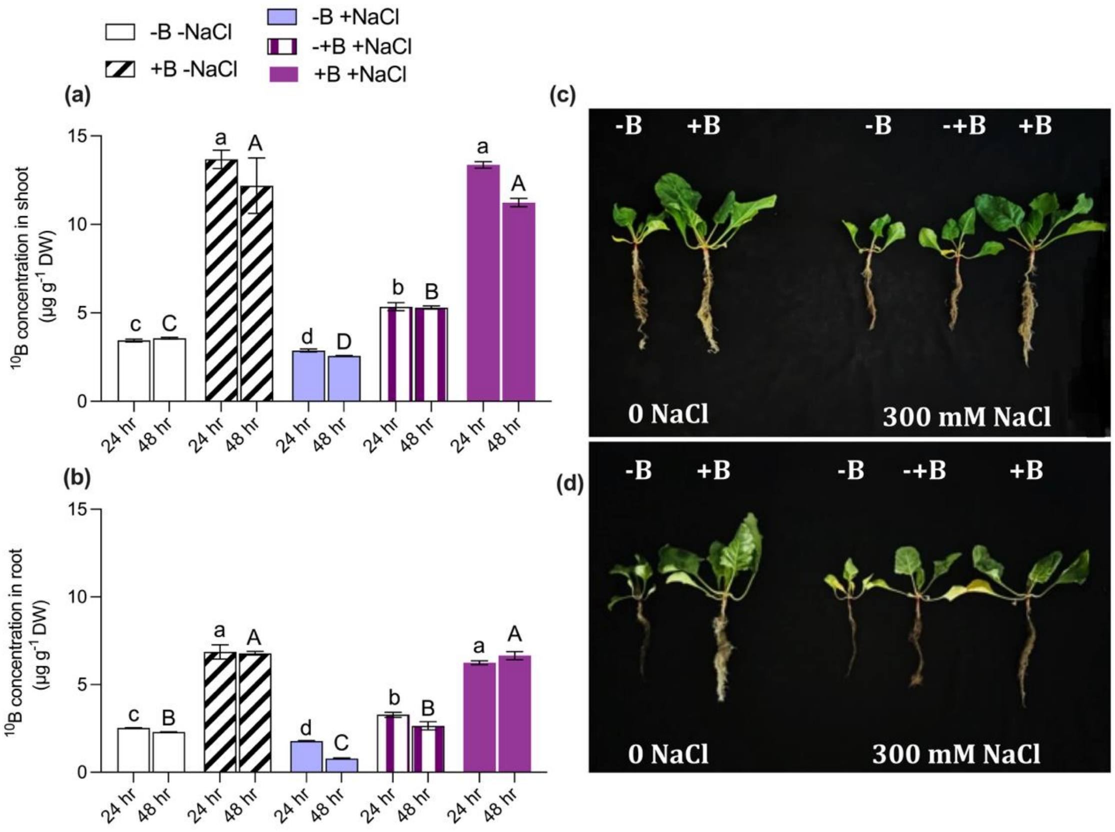


**Figure S2.** Boron ($^{10}$B) concentration in the shoot (a), root (b), phenotypic overview at 24 hours (hr) (c) and 48 hours (d) of sugar beet plants under different $^{10}$B treatment conditions with 0 and 300 mM NaCl salt stress. Sugar beet plants were cultivated in Hoagland's nutrient solution for 14 days with **1$^{st}$** (-B; 0.25 µM $^{10}$B), **2$^{nd}$** (-B; 0.25 µM $^{10}$B) and **3$^{rd}$** (+B; 25 µM $^{10}$B) for 14 days. Then, after up-salting, sugar beet plants were observed at 24 hr and 48 hr with the following treatment combinations: **1$^{st}$** (-B; 0.25 µM $^{10}$ B+0/300 mM NaCl), **2$^{nd}$** (-+B; 25 µM $^{10}$ B+0/300 mM NaCl) and **3$^{rd}$** (+B; 25 µM $^{10}$ B+ 0/300 mM NaCl). Values are means ± SD with 3 biological replicates ($n$ = 3) at $p$ < 0.05 (Tukey's HSD test).

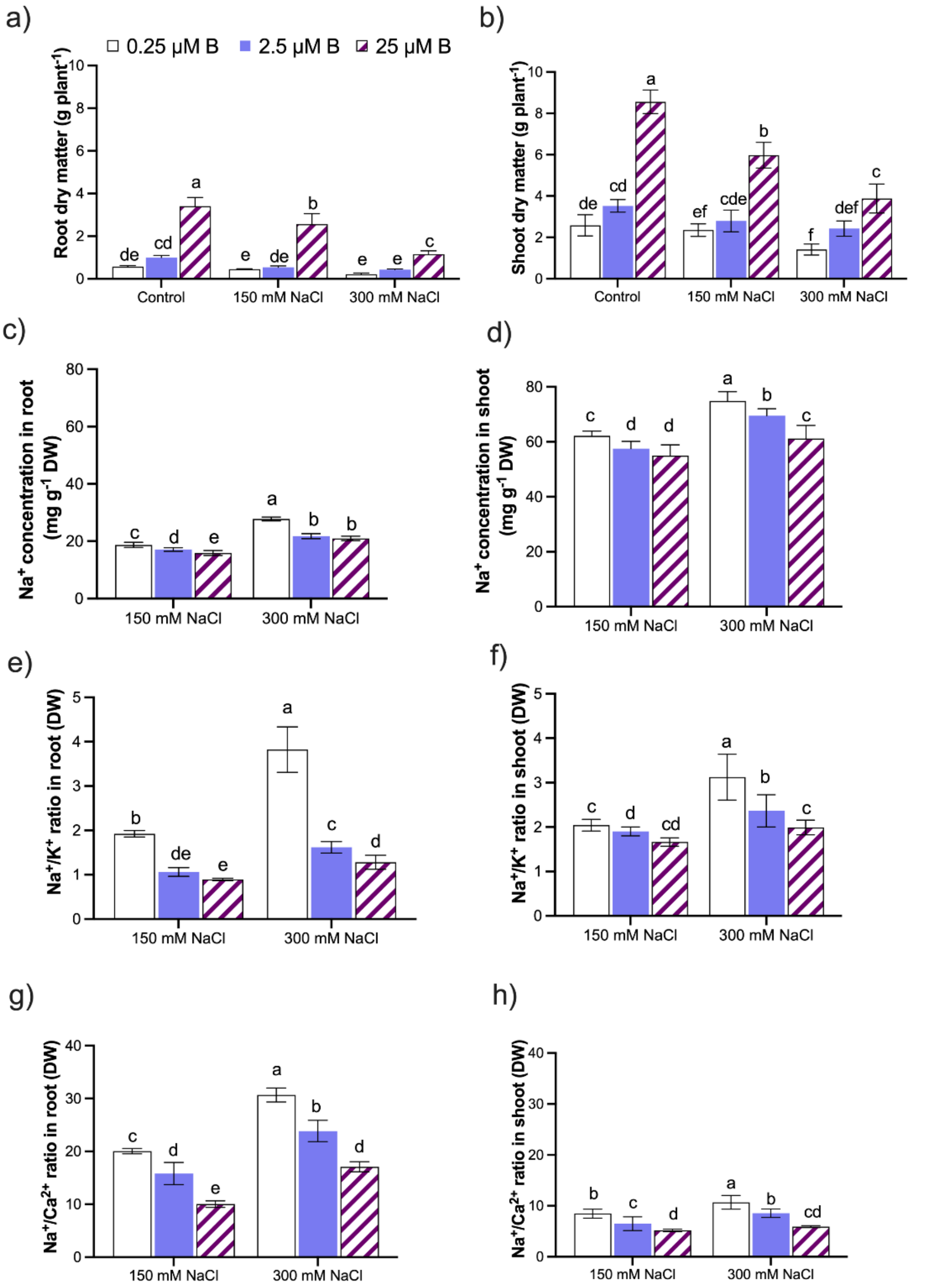


**Figure S3.** 1 Root, shoot dry matter (a, b), concentrations of $Na^+$ (c, d), the ratio of $Na^+/K^+$ (e, f), $Na^+/Ca^{2+}$ (g, h) in the root and shoot under different levels of salt-grown sugar beet plants at different boron conditions. Sugar beet plants were cultivated in Hoagland's nutrient solution for 28 days with 0.25 μM, 2.5 μM, and 25 μM $^{10}B$ conditions. Then, NaCl stress conditions (0, 150, 300 mM NaCl) were applied for another 14 days. Values are means ± SD with 5 biological replicates ($n$ = 5) at $p < 0.05$ (Tukey's HSD test).

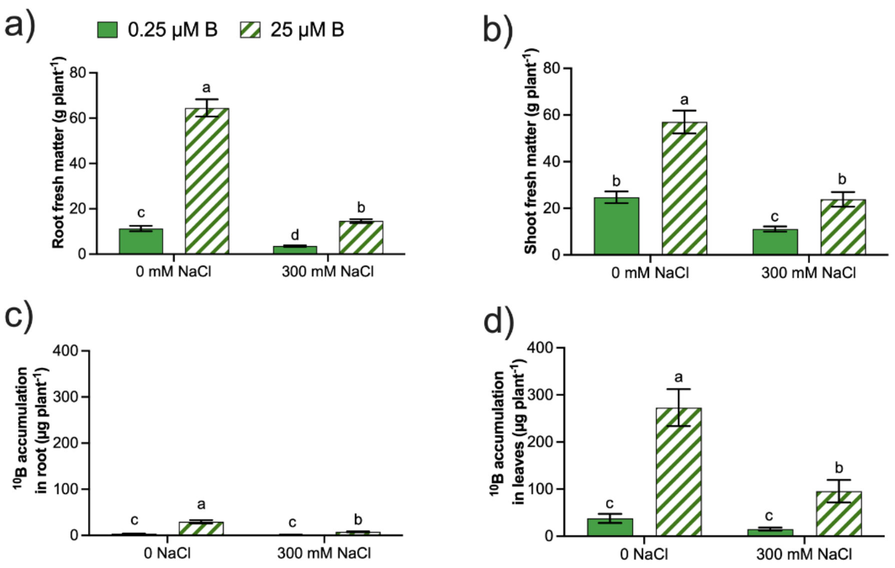


**Figure S4.** 4 Root, shoot fresh matter (a, b), accumulation of boron ($^{10}B$) (c, d) in the root, and shoot of high salt-grown sugar beet plants at low and adequate boron treatment. Sugar beet plants were cultivated in Hoagland's nutrient solution for 28 days at LB and AB conditions. Then, NaCl stress conditions (0/300 mM NaCl) for another 14 days, containing LB (0.25 µM $^{10}B$) and AB (25 µM $^{10}B$). Values are means ± SD with 4 biological replicates ($n$ = 4) at $p$ < 0.05 (Tukey's HSD test).

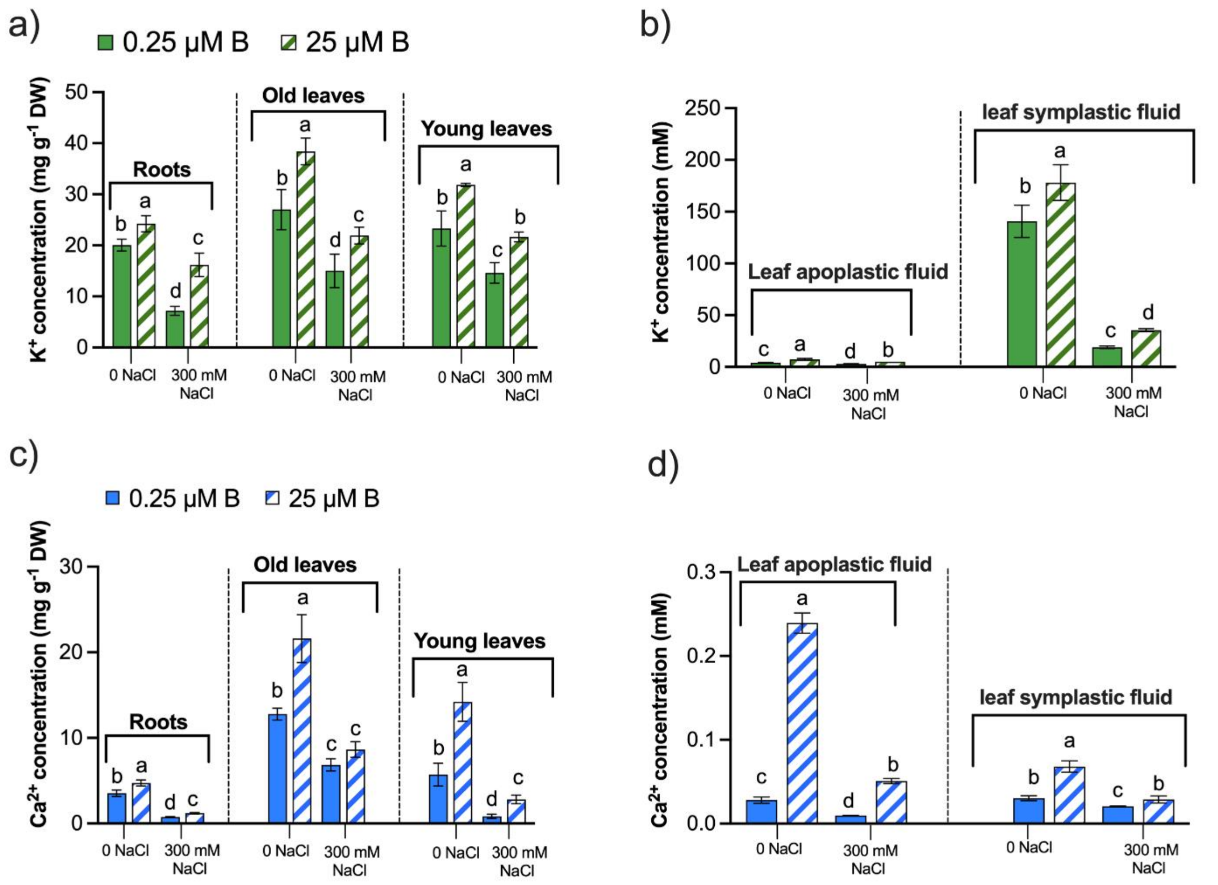


**Figure S5.** Quantification of $K^+$ (a, b), and $Ca^{2+}$ (c, d) concentrations in the roots, young and old leaves, leaf apoplastic and symplastic fluids of sugar beet plants under low (0.25 µM $^{10}B$; LB) and adequate (25 µM $^{10}B$; AB) levels of boron after NaCl stress (0 mM NaCl/300 mM NaCl). Sugar beet plants were cultivated in Hoagland's nutrient solution for 28 days at LB and AB conditions. Then, NaCl stress conditions (0 /300 mM NaCl) were applied for another 14 days, containing LB and AB. Values are means ± SD with 4 biological replicates ($n$ = 4) at $p$ < 0.05 (Tukey's HSD test).

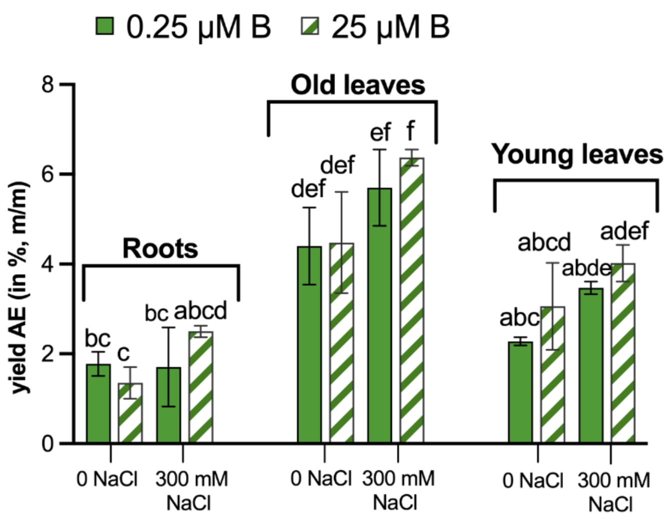


**Figure S6.** Yields of water-soluble fractions from the roots and leaves of *Beta vulgaris* under different salt and boron conditions.

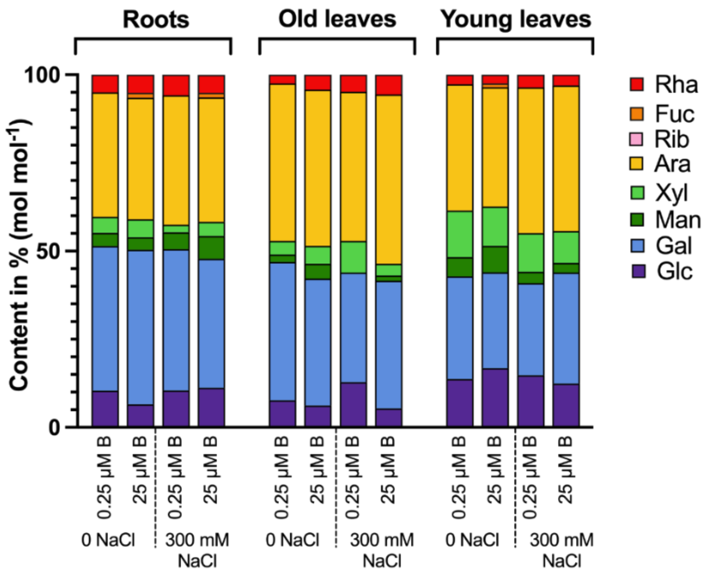


**Figure S7.** Neutral monosaccharide composition of water-soluble fractions from the roots and leaves of *Beta vulgaris* under different salt and boron conditions.

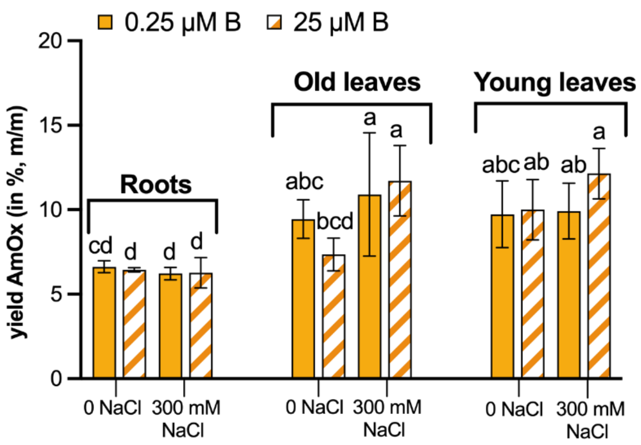


**Figure S8.** Yields of the pectic oxalate fraction from the roots and leaves of *Beta vulgaris* under different salt and boron conditions.

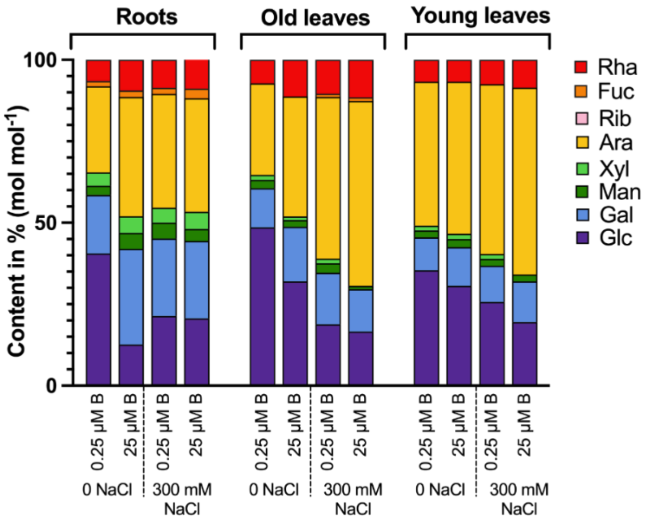


**Figure S9.** Neutral monosaccharide composition of the pectic oxalate fraction from the roots and leaves of *Beta vulgaris* under different salt and boron conditions.

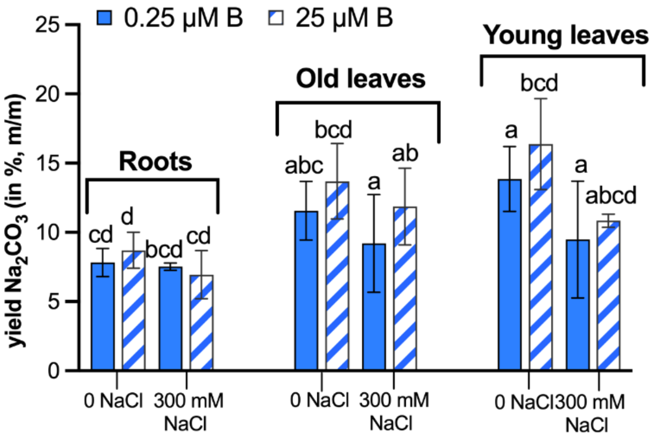


**Figure S10.** Yields of pectic $Na_2CO_3$ fractions from the roots and leaves of *Beta vulgaris* under different salt and boron conditions.

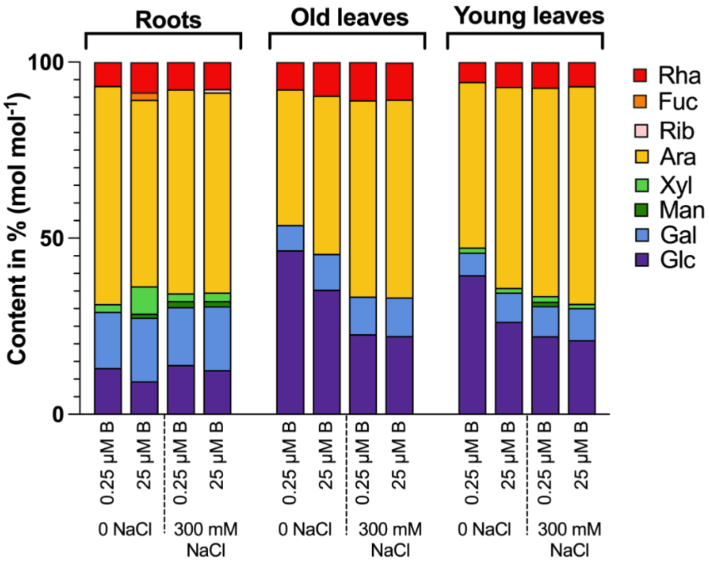


**Figure S11.** Neutral monosaccharide composition of pectic $Na_2CO_3$ fractions from the roots and leaves of *Beta vulgaris* under different salt and boron conditions.

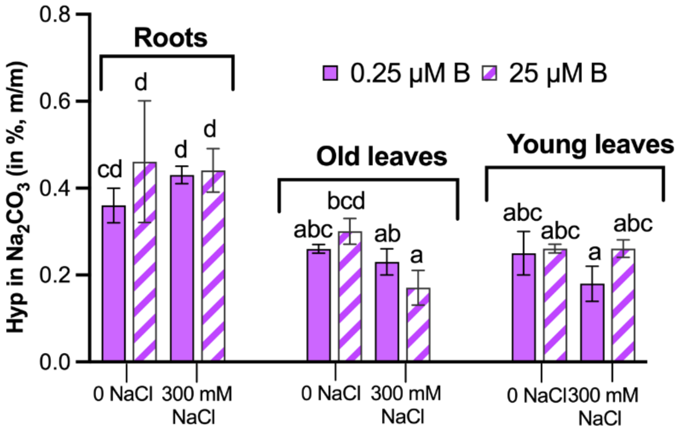


**Figure S12.** Amounts of Hyp in $Na_2CO_3$ fractions from the roots and leaves of *Beta vulgaris* under different salt and boron conditions.

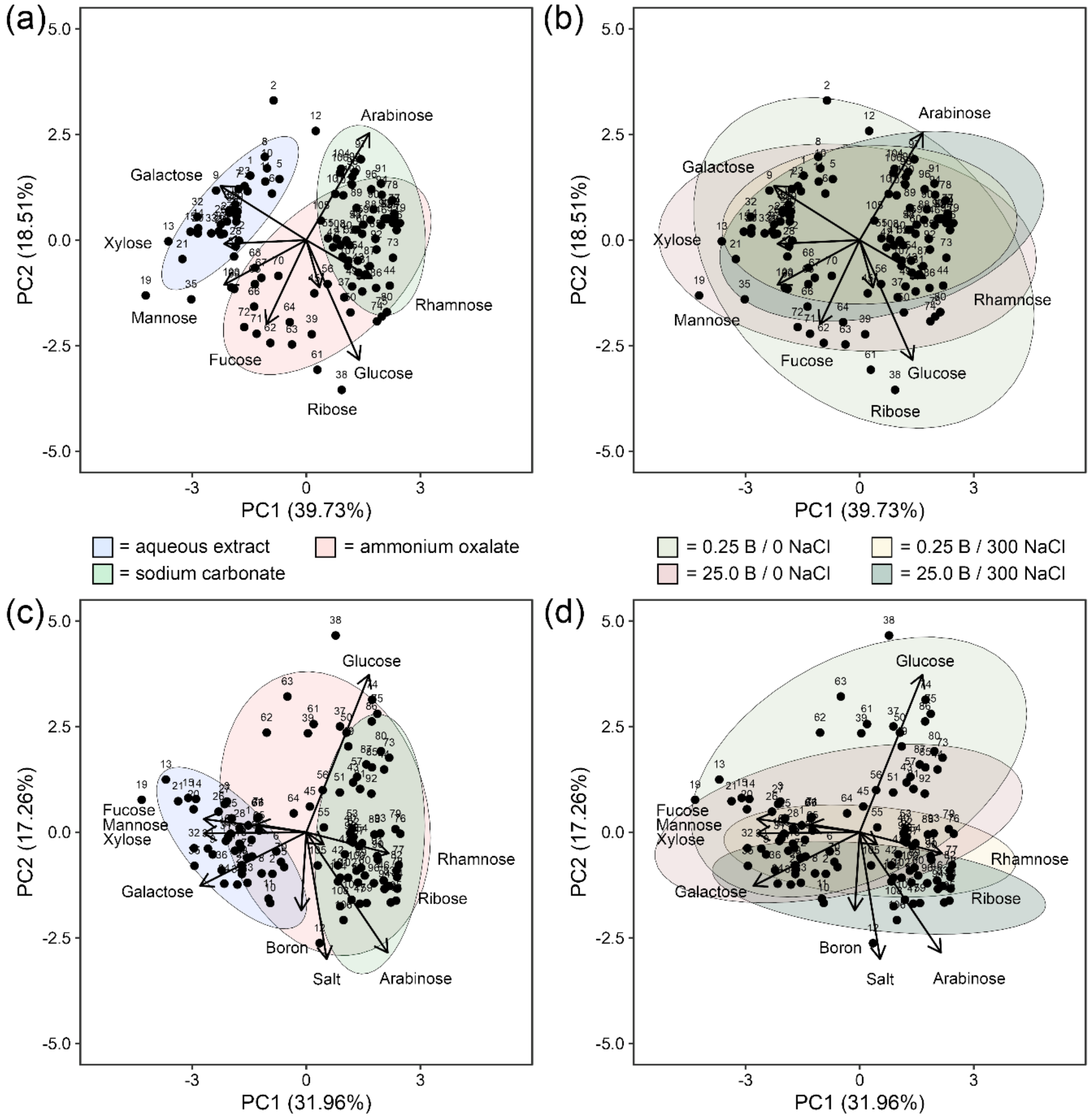


**Figure S13.** Principal component analysis of monosaccharide composition of the different cell wall fractions (aqueous extract, ammonium oxalate, sodium carbonate). (a, b) Plots showing PC1 vs. PC2 for the analysis with monosaccharide compositional values as loading vectors. (a) Plots with colored clusters for each type of the extract with ellipses representing the confidence intervals of 0.95. (b) Plots with colored clusters for each condition with ellipses representing the confidence intervals of 0.95. (c, d) Plots showing PC1 vs. PC2 for the analysis with monosaccharide compositional and conditional values (boron and salt concentrations) as loading vectors. (c) Plots with colored clusters for each type of extracts with ellipses representing the confidence intervals of 0.95. (d) Plots with colored clusters for each condition with ellipses representing the confidence intervals of 0.95.

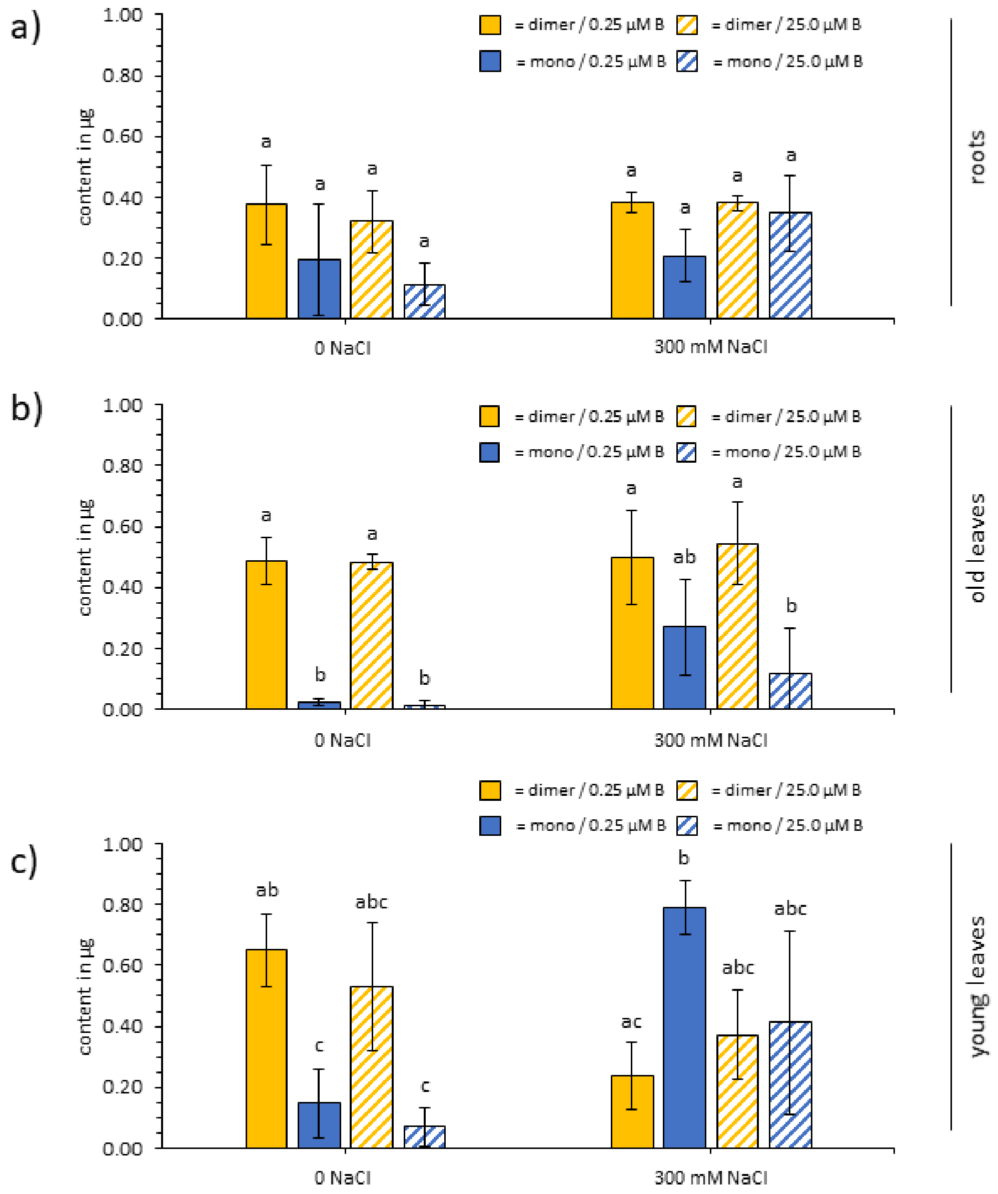


**Figure S14.** Contents of RG-II monomers and dimers in roots, old leaves and young leaves of *Beta vulgaris* grown under different treatments. Values are determined by quantification of polyacrylamide gels (n=3).

**Table S1a.** Neutral monosaccharide composition of the aqueous fractions from **roots** of *Beta vulgaris* grown under different salt and boron conditions in % (mol mol$^{-1}$; n=3; tr: trace value < 1 %).

| Neutral monosaccharide | 0.25 µM $^{10}$B 0 mM NaCl | 25 µM $^{10}$B 0 mM NaCl | 0.25 µM $^{10}$B 300 mM NaCl | 25 µM $^{10}$B 300 mM NaCl |
|---|---|---|---|---|
| PCA id | 25, 26, 27 | 28, 29, 30 | 31, 32, 33 | 34, 35, 36 |
| Rha | 5.0 ± 0.4 | 5.1 ± 0.2 | 5.8 ± 0.7 | 5.1 ± 0.2 |
| Fuc | tr | 1.4 ± 0.7 | tr | 1.3 ± 0.6 |
| Rib | - | - | - | tr |
| Ara | 35.3 ± 2.5 | 34.5 ± 3.4 | 36.7 ± 3.5 | 35.3 ± 2.9 |
| Xyl | 4.5 ± 0.8 | 5.1 ± 1.1 | 2.2 ± 0.1 | 4.0 ± 1.0 |
| Man | 3.8 ± 0.4 | 3.5 ± 0.3 | 4.8 ± 0.7 | 6.5 ± 1.7 |
| Gal | 41.0 ± 2.1 | 43.8 ± 4.7 | 40.0 ± 3.2 | 36.6 ± 2.9 |
| Glc | 10.4 ± 1.7 | 6.6 ± 2.5 | 10.5 ± 6.2 | 11.2 ± 0.9 |

**Table S1b.** Neutral monosaccharide composition of the aqueous fractions from **old leaves** of *Beta vulgaris* grown under different salt and boron conditions in % (mol mol$^{-1}$; n=3; tr: trace value < 1 %).

| Neutral monosaccharide | 0.25 µM $^{10}$B 0 mM NaCl | 25 µM $^{10}$B 0 mM NaCl | 0.25 µM $^{10}$B 300 mM NaCl | 25 µM $^{10}$B 300 mM NaCl |
|---|---|---|---|---|
| PCA id | 1, 2, 3 | 4, 5, 6 | 7, 8, 9 | 10, 11, 12 |
| Rha | 2.5 ± 2.2 | 4.2 ± 0.6 | 4.8 ± 0.6 | 5.6 ± 1.3 |
| Fuc | - | - | - | - |
| Rib | - | - | - | - |
| Ara | 44.6 ± 9.9 | 44.3 ± 4.8 | 42.3 ± 2.3 | 48.0 ± 5.0 |
| Xyl | 3.9 ± 3.4 | 5.1 ± 2.6 | 9.0 ± 0.2 | 3.3 ± 2.9 |
| Man | 2.1 ± 2.4 | 4.2 ± 1.6 | tr | 1.5 ± 1.4 |
| Gal | 39.2 ± 4.9 | 36.0 ± 1.4 | 31.1 ± 1.4 | 36.2 ± 3.7 |
| Glc | 7.7 ± 7.0 | 6.2 ± 1.7 | 12.8 ± 1.8 | 5.4 ± 4.7 |

**Table S1c.** Neutral monosaccharide composition of the aqueous fractions from **young leaves** of *Beta vulgaris* grown under different salt and boron conditions in % (mol mol$^{-1}$; n=3; tr: trace value < 1 %).

| Neutral monosaccharide | 0.25 µM $^{10}$B 0 mM NaCl | 25 µM $^{10}$B 0 mM NaCl | 0.25 µM $^{10}$B 300 mM NaCl | 25 µM $^{10}$B 300 mM NaCl |
|---|---|---|---|---|
| PCA id | 13, 14, 15 | 16, 17, 18 | 19, 20, 21 | 22, 23, 24 |
| Rha | 2.7 ± 0.5 | 2.5 ± 0.3 | 3.6 ± 0.4 | 3.0 ± 0.2 |
| Fuc | tr | 1.0 ± 1.3 | tr | tr |
| Rib | - | - | - | tr |
| Ara | 35.8 ± 2.6 | 33.9 ± 0.5 | 41.3 ± 1.6 | 41.3 ± 0.2 |
| Xyl | 13.2 ± 0.7 | 11.1 ± 2.4 | 11.0 ± 0.3 | 9.1 ± 1.5 |
| Man | 5.4 ± 1.8 | 7.5 ± 1.1 | 3.2 ± 0.5 | 2.7 ± 0.6 |
| Gal | 29.2 ± 1.8 | 27.2 ± 4.3 | 26.1 ± 1.8 | 31.5 ± 2.2 |
| Glc | 13.7 ± 1.9 | 16.8 ± 1.0 | 14.8 ± 0.8 | 12.4 ± 2.0 |

**Table S2a.** Neutral monosaccharide composition of the ammonium oxalate fractions from **roots** of *Beta vulgaris* grown under different salt and boron conditions in % (mol mol$^{-1}$; n=3; tr: trace value < 1 %).

| **Neutral monosaccharide** | **0.25 µM $^{10}$B 0 mM NaCl** | **25 µM $^{10}$B 0 mM NaCl** | **0.25 µM $^{10}$B 300 mM NaCl** | **25 µM $^{10}$B 300 mM NaCl** |
|---|---|---|---|---|
| PCA id | 61, 62, 63 | 64, 65, 66 | 67, 68, 69 | 70, 71, 72 |
| Rha | 6.5 ± 0.9 | 9.4 ± 0.3 | 8.6 ± 0.2 | 8.8 ± 1.5 |
| Fuc | 1.6 ± 0.9 | 2.0 ± 0.5 | 1.9 ± 0.6 | 3.0 ± 1.5 |
| Rib | tr | tr | tr | tr |
| Ara | 26.4 ± 2.6 | 36.6 ± 4.7 | 34.9 ± 2.5 | 34.8 ± 3.5 |
| Xyl | 4.1 ± 1.2 | 5.1 ± 0.8 | 4.6 ± 1.4 | 5.3 ± 1.2 |
| Man | 2.9 ± 0.5 | 4.9 ± 0.4 | 4.8 ± 0.7 | 3.7 ± 0.1 |
| Gal | 17.9 ± 1.3 | 29.4 ± 3.9 | 23.8 ± 1.9 | 23.8 ± 2.5 |
| Glc | 40.6 ± 6.0 | 12.6 ± 3.3 | 21.4 ± 4.0 | 20.6 ± 6.9 |

**Table S2b.** Neutral monosaccharide composition of the ammonium oxalate fractions from **old leaves** of *Beta vulgaris* grown under different salt and boron conditions in % (mol mol$^{-1}$; n=3; tr: trace value < 1 %).

| **Neutral monosaccharide** | **0.25 µM $^{10}$B 0 mM NaCl** | **25 µM $^{10}$B 0 mM NaCl** | **0.25 µM $^{10}$B 300 mM NaCl** | **25 µM $^{10}$B 300 mM NaCl** |
|---|---|---|---|---|
| PCA id | 37, 38, 39 | 40, 41, 42 | 43, 44, 45 | 46, 47, 48 |
| Rha | 7.2 ± 1.6 | 11.2 ± 1.7 | 10.4 ± 0.6 | 11.5 ± 1.1 |
| Fuc | tr | tr | 1.1 ± 0.9 | 1.1 ± 1.0 |
| Rib | - | - | - | - |
| Ara | 28.1 ± 9.4 | 36.9 ± 0.9 | 49.6 ± 4.8 | 56.8 ± 6.0 |
| Xyl | 1.5 ± 1.5 | 1.1 ± 1.8 | 1.4 ± 1.3 | tr |
| Man | 2.6 ± 0.7 | 2.0 ± 2.0 | 2.9 ± 0.2 | 1.0 ± 0.9 |
| Gal | 12.0 ± 4.1 | 16.8 ± 3.3 | 15.9 ± 2.8 | 13.0 ± 3.0 |
| Glc | 48.6 ± 15.6 | 32.0 ± 8.3 | 18.8 ± 4.7 | 16.6 ± 3.5 |

**Table S2c.** Neutral monosaccharide composition of the ammonium oxalate fractions from **young leaves** of *Beta vulgaris* grown under different salt and boron conditions in % (mol mol$^{-1}$; n=3; tr: trace value < 1 %).

| **Neutral monosaccharide** | **0.25 µM $^{10}$B 0 mM NaCl** | **25 µM $^{10}$B 0 mM NaCl** | **0.25 µM $^{10}$B 300 mM NaCl** | **25 µM $^{10}$B 300 mM NaCl** |
|---|---|---|---|---|
| PCA id | 49, 50, 51 | 52, 53, 54 | 55, 56, 57 | 58, 59, 60 |
| Rha | 6.7 ± 0.5 | 6.7 ± 0.1 | 7.5 ± 0.3 | 8.5 ± 1.2 |
| Fuc | tr | tr | tr | tr |
| Rib | tr | tr | tr | tr |
| Ara | 44.2 ± 5.5 | 46.7 ± 5.1 | 52.1 ± 1.7 | 57.4 ± 2.7 |
| Xyl | 1.4 ± 0.4 | 1.6 ± 0.5 | 1.5 ± 0.4 | tr |
| Man | 2.2 ± 0.7 | 2.5 ± 0.6 | 2.1 ± 0.3 | 2.1 ± 0.1 |
| Gal | 10.1 ± 2.3 | 11.9 ± 3.3 | 11.1 ± 0.3 | 12.5 ± 1.1 |
| Glc | 35.4 ± 8.8 | 30.6 ± 8.3 | 25.7 ± 1.2 | 19.5 ± 3.9 |

**Table S3a.** Neutral monosaccharide composition of the sodium carbonate fractions from **roots** of *Beta vulgaris* grown under different salt and boron conditions in % (mol $mol^{-1}$; n=3; tr: trace value < 1 %).

| Neutral monosaccharide | 0.25 µM $^{10}$B 0 mM NaCl | 25 µM $^{10}$B 0 mM NaCl | 0.25 µM $^{10}$B 300 mM NaCl | 25 µM $^{10}$B 300 mM NaCl |
|---|---|---|---|---|
| PCA id | 97, 98, 99 | 100, 101, 102 | 103, 104, 105 | 106, 107, 108 |
| Rha | 6.7 ± 0.7 | 8.6 ± 2.8 | 7.7 ± 0.8 | 7.6 ± 2.1 |
| Fuc | tr | 2.1 ± 2.2 | tr | tr |
| Rib | - | - | - | 1.0 ± 0.7 |
| Ara | 62.0 ± 1.0 | 52.9 ± 7.6 | 57.9 ± 0.7 | 56.8 ± 6.1 |
| Xyl | 2.1 ± 0.3 | 7.8 ± 8.4 | 2.2 ± 0.4 | 2.4 ± 0.5 |
| Man | tr | 1.2 ± 1.2 | 1.7 ± 0.2 | 1.5 ± 0.5 |
| Gal | 16.0 ± 0.8 | 18.0 ± 4.5 | 16.4 ± 1.5 | 18.1 ± 0.7 |
| Glc | 13.2 ± 0.3 | 9.4 ± 1.8 | 14.1 ± 2.0 | 12.6 ± 2.5 |

**Table S3b.** Neutral monosaccharide composition of the sodium carbonate fractions from **old leaves** of *Beta vulgaris* grown under different salt and boron conditions in % (mol $mol^{-1}$; n=3; tr: trace value < 1 %).

| Neutral monosaccharide | 0.25 µM $^{10}$B 0 mM NaCl | 25 µM $^{10}$B 0 mM NaCl | 0.25 µM $^{10}$B 300 mM NaCl | 25 µM $^{10}$B 300 mMNaCl |
|---|---|---|---|---|
| PCA id | 73, 74, 75 | 76, 77, 78 | 79, 80, 81 | 82, 83, 84 |
| Rha | 7.7 ± 0.7 | 9.5 ± 1.4 | 10.8 ± 0.9 | 10.5 ± 1.1 |
| Fuc | tr | tr | - | tr |
| Rib | tr | tr | tr | tr |
| Ara | 38.5 ± 6.4 | 44.9 ± 8.0 | 55.7 ± 3.8 | 56.2 ± 2.1 |
| Xyl | tr | tr | tr | tr |
| Man | tr | tr | tr | tr |
| Gal | 7.2 ± 1.1 | 10.2 ± 1.9 | 10.7 ± 0.1 | 10.9 ± 0.3 |
| Glc | 46.6 ± 7.9 | 35.4 ± 10.2 | 22.8 ± 4.8 | 22.3 ± 2.8 |

**Table S3c.** Neutral monosaccharide composition of the sodium carbonate fractions from **young leaves** of *Beta vulgaris* grown under different salt and boron conditions in % (mol $mol^{-1}$; n=3; tr: trace value < 1 %).

| Neutral monosaccharide | 0.25 µM $^{10}$B 0 mM NaCl | 25 µM $^{10}$B 0 mM NaCl | 0.25 µM $^{10}$B 300 mM NaCl | 25 µM $^{10}$B 300 mM NaCl |
|---|---|---|---|---|
| PCA id | 85, 86, 87 | 88, 89, 90 | 91, 92, 93 | 94, 95, 96 |
| Rha | 5.6 ± 1.0 | 7.0 ± 0.9 | 7.2 ± 1.2 | 6.8 ± 1.7 |
| Fuc | tr | tr | tr | tr |
| Rib | tr | tr | - | tr |
| Ara | 47.0 ± 5.0 | 57.1 ± 6.7 | 59.2 ± 2.7 | 61.8 ± 0.5 |
| Xyl | 1.5 ± 0.8 | 1.3 ± 0.1 | 1.6 ± 0.3 | 1.2 ± 0.2 |
| Man | tr | tr | 1.2 ± 1.1 | tr |
| Gal | 6.4 ± 1.5 | 8.3 ± 0.9 | 8.6 ± 0.5 | 9.1 ± 0.1 |
| Glc | 39.5 ± 7.1 | 26.3 ± 8.4 | 22.2 ± 5.4 | 21.1 ± 1.8 |
